# The effects of shared, depression-specific, and anxiety-specific internalizing symptoms on negative and neutral episodic memories following post-learning sleep

**DOI:** 10.3758/s13415-024-01209-5

**Published:** 2024-08-13

**Authors:** Xinran Niu, Mia F. Utayde, Kristin E. G. Sanders, Tony J. Cunningham, Guangjian Zhang, Elizabeth A. Kensinger, Jessica D. Payne

**Affiliations:** 1 Department of Psychology, University of Notre Dame, E466 Corbett Family Hall, Notre Dame, IN 46556 USA; 2 The Center for Sleep & Cognition, Harvard Medical School & Beth Israel Deaconess Medical Center, Boston, MA USA; 3 Department of Psychology and Neuroscience, Boston College, Chestnut Hill, MA USA

**Keywords:** Memory consolidation, Emotional memory, Sleep, Depression, Anxiety, Bifactor

## Abstract

Emotional memory bias is a common characteristic of internalizing symptomatology and is enhanced during sleep. The current study employs bifactor S-1 modeling to disentangle depression-specific anhedonia, anxiety-specific anxious arousal, and the common internalizing factor, general distress, and test whether these internalizing symptoms interact with sleep to influence memory for emotional and neutral information. Healthy adults (*N* = 281) encoded scenes featuring either negative objects (e.g., a vicious looking snake) or neutral objects (e.g., a chipmunk) placed on neutral backgrounds (e.g., an outdoor scene). After a 12-hour period of daytime wakefulness (*n* = 140) or nocturnal sleep (*n* = 141), participants judged whether objects and backgrounds were the same, similar, or new compared with what they viewed during encoding. Participants also completed the mini version of the Mood and Anxiety Symptom Questionnaire. Higher anxious arousal predicted worse memory across all stimuli features, but only after a day spent being awake—not following a night of sleep. No significant effects were found for general distress and anhedonia in either the sleep or wake condition. In this study, internalizing symptoms were not associated with enhanced emotional memory. Instead, memory performance specifically in individuals with higher anxious arousal was impaired overall, regardless of emotional valence, but this was only the case when the retention interval spanned wakefulness (i.e., not when it spanned sleep). This suggests that sleep may confer a protective effect on general memory impairments associated with anxiety.

**Supplementary Information:**

The online version contains supplementary material available at 10.3758/s13415-024-01209-5.

We remember emotionally arousing experiences more vividly than neutral experiences (Brown & Kulik, 1977; Holland & Kensinger, 2010; Kensinger, 2009). Although multiple factors contribute to this emotional memory bias, one factor involves the release of stress- and arousal-related neuromodulators (e.g., norepinephrine) at the time of encoding. These substances trigger an increase in attentional resources that help to mark emotional experiences as important at the time of encoding (Talmi, 2013; Tully & Bolshakov, 2010).

Emotional information that is “tagged” as salient at the encoding stage also is preferentially reactivated during memory consolidation, including, and perhaps especially, during sleep (Bennion, Mickley et al., 2015a, Bennion, Payne et al., 2015b, 2017; Kim & Payne, 2020; Payne & Kensinger, 2018). Rapid eye movement (REM) sleep, which is characterized by increased theta oscillations and acetylcholine levels—both of which increase the functional connectivity within the hippocampal-amygdala memory circuit—may be important for emotional memory consolidation (Bennion, Payne et al., 2015b; Bennion et al., 2017; Hutchison & Rathore, 2015). However, well-consolidated emotional memories likely depend on an interplay between REM sleep and Slow Wave Sleep (SWS), because the triple coupling of slow oscillations, sleep spindles, and sharp-wave/ripple complexes during SWS are critical for information flow from the hippocampus to neocortical networks (Cairney et al., 2015; Klinzing et al., 2019). Despite strong theoretical and some empirical support (Cunningham & Payne, 2017; Kim & Payne, 2020; Payne, Ellenbogen, et al., 2008a), recent meta-analyses suggest that post-learning sleep does not consistently enhance emotional over neutral memory across studies using different emotional memory tasks (Lipinska et al., 2019; Schäfer et al., 2020). Because these inconsistent results might be attributed to low statistical power and lack of standardization in measuring episodic emotional memory, high-powered replication efforts are needed to evaluate this sleep-related selectivity for the consolidation of emotional memories (Cunningham et al., 2022; Németh et al., 2023).

In addition to its role in memory consolidation, sleep impacts emotional processing more generally; poor sleep exacerbates mood disorders, anxiety disorders, and internalizes symptomatology (Niu et al., 2021, 2023; Riemann et al., 2001). Internalizing symptoms are characterized by inner-directed negative emotions, including sadness, loneliness, and worry, which represent a broad range of depression and anxiety symptoms (Krueger & Markon, 2006; Park et al., 2020). Whereas REM sleep dysregulation is an important clinical marker of depression, including increased REM duration, decreased REM latency (i.e., the amount of time between sleep onset and first REM period), and increased REM density (i.e., the number of eye movements for each REM period; Palagini et al., 2013), sleep disturbances related to internalizing symptoms extend beyond REM sleep. Reductions in slow-wave sleep (SWS) duration and slow-wave amplitude also contribute to more severe internalizing symptoms, particularly for anxiety (Ben Simon et al., 2020; Chellappa & Aeschbach, 2022). Because REM sleep may preferentially benefit the consolidation of events tagged as emotionally salient during wakefulness (Bennion, Mickley et al., 2015a, Bennion, Payne et al 2015b, 2017; Kim & Payne, 2020; Payne & Kensinger, 2018), this altered sleep structure, characterized by both prolonged REM sleep and diminished SWS, might contribute to negative memory bias in individuals with higher internalizing symptoms (Harrington, Johnson et al., 2018a; Harrington et al., 2023).

In support of this, meta-analyses show that individuals with higher depression and anxiety symptoms often demonstrate increased memory for negative compared with neutral stimuli, both immediately after encoding and across brief waking delays (e.g., less than half an hour; James et al., 2021; Mitte, 2008). However, it remains unclear whether post-learning sleep further increases the effects of depression and anxiety on memory by further biasing the brain toward the consolidation of negative emotional memories.

Given the extensive comorbidity between depression and anxiety symptoms (Gorman, 1996; Kessler et al., 2008), previous research has advocated conceptualizing depression and anxiety as latent internalizing symptom dimensions as opposed to distinct categorical disorders (Hankin et al., 2016; Kessler et al., 2008). The Tripartite Model of Anxiety and Depression (Clark & Watson, 1991) divided internalizing symptoms into three dimensions: a) general distress that is shared between depression and anxiety; b) anhedonia that is unique to depression; and c) anxious arousal that is unique to anxiety (Anderson & Hope, 2008). Common, depression-specific, and anxiety-specific internalizing symptoms have been shown to have differential effects on cognitive performance (Bowman et al., 2019; Peterson et al., 2022). The current study attempts to connect sleep research with this contemporary understanding of clinical symptomology, examining whether common and specific dimensions of internalizing symptoms interact with sleep to predict biased emotional memory consolidation.

## Internalizing symptoms and emotional memory

The current body of research is mixed regarding whether depression and anxiety individually predict memory performance for negative information. While some studies show that both depressed (Everaert et al., 2014; Fattahi Asl et al., 2015; Hakamata et al., 2022; Miles et al., 2004) and anxious individuals (Eden et al., 2015; Hakamata et al., 2022; Miles et al., 2004) recall and recognize more emotionally negative stimuli compared with neutral and positive ones, meta-analytic reviews indicate that depression contributes to increased negative emotional memory when the information is self-relevant (James et al., 2021), whereas anxiety-related memory biases are specifically toward threatening information (Herrera et al., 2017). However, given the high co-occurrence between depression and anxiety, it seems unlikely that they would independently predict emotional memory biases (Hankin et al., 2016).

Previous studies have examined the potential unique and shared contributions of depression and anxiety on emotional memory. Some that included both depression and anxiety in the same model found mixed results regarding whether depression and anxiety symptoms both uniquely and equally contributed to increased recognition: in this case for threat-related words associated with physical danger (Coles et al., 2007; Dowens & Calvo, 2003). Other studies evaluated the shared contributions of depression and anxiety by conducting diagnostic interviews to determine whether participants had only depression, only anxiety, or comorbid depression and anxiety (Gilboa-Schechtman et al., 2002; LeMoult & Joormann, 2012). These studies found that individuals with comorbid depression and anxiety are more likely to show increased recognition for angry facial expressions compared with healthy controls, although there are inconsistent findings regarding whether individuals with comorbid depression and anxiety recognize more angry faces than individuals with either depression or anxiety alone (Gilboa-Schechtman et al., 2002; LeMoult & Joormann, 2012).

One reason for these mixed findings could be that different cognitive deficits related to internalizing symptomatology may counterbalance or offset each other’s effects. Internalizing symptoms are associated with executive functioning-related deficits that affect sustained attention and working memory and with motivation-related deficits that affect cognitive control allocation (Grahek et al., 2019; Snyder & Hankin, 2019). Conversely, internalizing symptoms are associated with emotional regulation deficits that increase worry and rumination and prolong the duration of negative emotional processing in working memory (Niu et al., 2023; Niu & Snyder, 2022; Taylor & Snyder, 2021). As a result, it is possible that internalizing symptoms impair overall memory and cognitive performance more than they selectively impact emotional aspects of memory, which would lead to null effects of internalizing symptoms on emotional memory bias when measured behaviorally.

In general, numerous studies suggest that emotional memory bias is more pronounced in cases of comorbid depression and anxiety compared with either pure depression or pure anxiety (Coles et al., 2007; Dowens & Calvo, 2003; Gilboa-Schechtman et al., 2002; LeMoult & Joormann, 2012). More studies are needed to determine whether this is simply because the comorbidity of depression and anxiety reflects a greater degree of distress and dysfunction or because different mechanisms underlie the shared internalizing symptom component versus the unique depression and anxiety components.

### Sleep and emotional memory

Although many studies find that post-learning sleep benefits overall memory performance (Baran et al., 2012; Bolinger et al., 2018; Göder et al., 2015; Hu et al., 2006; Kurz et al., 2019; Morgenthaler et al., 2014; Payne, Ellenbogen et al., 2008a; Prehn-Kristensen et al., 2009, 2013), not all find more sleep-related enhancement for emotional memory than neutral memory (Ashton et al., 2019; Baran et al., 2012; Bolinger et al., 2019; Cox et al., 2018; Jones et al., 2016; Wiesner et al., 2015). One emotional memory paradigm that shows more consistent results employs the emotional memory trade-off task. This task evaluates recognition memory for different components of scenes containing a negative or neutral object placed on a neutral background (Cunningham et al., 2014; Davidson et al., 2021; Denis et al., 2022; Payne et al., 2012; Payne, Ellenbogen et al., 2008a; Payne, Stickgold et al., 2008b; Payne & Kensinger, 2011). These studies typically find that participants who sleep recognize more negative objects than neutral objects compared with their backgrounds, confirming sleep-related emotional memory trade-off effects (Cunningham et al., 2014; Denis et al., 2022; Payne et al., 2012, Payne, Ellenbogen et al., 2008a, Payne, Stickgold et al., 2008b; Payne & Kensinger, 2011). Thus, sleep may prioritize memories for emotionally salient objects at the expense of all other information presented at encoding.

It also is important to note that most previous studies examining sleep-dependent effects on emotional memory were conducted in overnight laboratory experimental designs with relatively small sample sizes, and participants from these studies were limited to healthy, young adults and primarily college students (Ashton et al., 2019; Bolinger et al., 2019; Cox et al., 2018; Cunningham et al., 2014; Morgenthaler et al., 2014; Payne, Ellenbogen et al., 2008a, Payne, Stickgold et al., 2008b, 2012; Payne & Kensinger, 2011; Wiesner et al., 2015). Therefore, although these laboratory overnight studies exert strong experimental control, their findings are hindered by low statistical power and poor generalizability to detect true effects in the broader population (Davidson et al., 2021). More recent studies have used online tools to recruit a larger and broader population sample (Denis et al., 2022; Niu et al., 2024), which have provided clearer, higher-powered behavioral evidence for sleep-related emotional memory enhancements (Cunningham et al., 2014; Denis et al., 2022; Payne, Ellenbogen et al., 2008a, Payne, Stickgold et al., 2008b, 2012; Payne & Kensinger, 2011).

### Internalizing symptoms, sleep, and emotional memory

Recent studies have begun to examine how internalizing symptoms might interact with sleep to influence emotional memory consolidation. One study found that individuals reporting higher depression symptoms recognized more negative images compared with those with lower symptoms after REM-rich sleep, although not significantly (Harrington, Johnson et al., 2018a). Another study found that total sleep deprivation impaired memory performance for neutral and negative images among individuals with higher depression symptoms but not those with lower symptoms (Harrington, Nedberge et al., 2018b). Both studies suggested more robust sleep-related effects on emotional memory among individuals with high versus low depression symptoms, but little is known about whether these effects are restricted to depression-specific symptoms or could be extended to common internalizing and anxiety-specific symptoms as well.

### The current study

In the current study, we used data from an online experiment that has a relatively large sample size (*n* = 281) of young and middle-aged adults (Denis et al., 2022). The first goal of the study was to use bifactor S-1 modeling to disentangle common (i.e., general distress), depression-specific (i.e., anhedonia), and anxiety-specific (i.e., anxious arousal) internalizing symptom dimensions as proposed by the Tripartite Model of Anxiety and Depression (Clark & Watson, 1991). Bifactor models extract one single transdiagnostic construct underlying the broad internalizing symptoms from more narrowly defined subdomain constructs that are unique to depression and anxiety (Reise, 2012). However, many studies that applied bifactor modeling also found anomalous results, such as nonsignificant factor loadings and irregular loading patterns (Eid et al., 2017; Greene et al., 2019). An alternative, bifactor S-1 model has been proposed to address these psychometric issues by selecting one specific factor as a reference domain to give unambiguous meaning to the common factor (Burke & Johnston, 2020; Burns et al., 2020; Heinrich et al., 2020). In the current study, we used bifactor S-1 models to load items that measured general distress onto a common internalizing factor. This common internalizing factor represented the shared variance between a depression-specific anhedonia factor and an anxiety-specific anxious arousal factor. We hypothesized that bifactor S-1 models would fit our data well as bifactors generally have superior fit in models that attempt to separate common and specific dimensions of internalizing symptoms (Brodbeck et al., 2011; den Hollander-Gijsman et al., 2012; Fassett-Carman et al., 2020).

The second goal was to evaluate the effects of common and specific dimensions of internalizing symptoms—measured by the bifactor S-1 model—on emotional memory consolidation. Given that memory deficits associated with internalizing symptoms may grow exponentially during the 12-h retention interval in the current study (Snyder & Hankin, 2019), we hypothesized that higher internalizing symptoms would be associated with worse recognition memory across all types of stimuli. In addition, we hypothesized that if internalizing symptoms were associated with rating negative scenes as more negative and arousing than neutral scenes (i.e., more differentiated valence and arousal ratings), internalizing symptoms also would predict better recognition memory for negative objects compared with both neutral objects from separate scenes and paired neutral backgrounds from the same scenes. Alternatively, participants with higher internalizing symptoms may not be able to differentiate negative from neutral information, because they tend to perceive ambiguous information as negative and arousing (Ito et al., 2017), which would lead to equivalent memory for components of negative and neutral scenes.

The third goal was to test whether sleep interacted with common and specific internalizing symptoms measured by the bifactor S-1 model to predict emotional memory consolidation. First, because many studies have demonstrated sleep-related benefits on overall memory performance (Baran et al., 2012; Bolinger et al., 2018; Göder et al., 2015; Kurz et al., 2019; Morgenthaler et al., 2014; Prehn-Kristensen et al., 2009, 2013), we hypothesized that the relationship between internalizing symptoms and worsened recognition memory across all types of scene components would be weaker after post-learning sleep compared with wakefulness. Second, we hypothesized that if internalizing symptoms were associated with more differentiated valence and arousal ratings between negative and neutral scenes, the effects of internalizing symptoms on enhanced emotional memory would be stronger after post-learning sleep.

## Methods

### Participants

A total of 554 eligible participants were recruited online via Prolific (https://www.prolific.co) in March 2021. Eligibility criteria specified that participants 1) be 18–59 years of age, 2) be currently residing in the United States, 3) be fluent in English, 4) have scored at least 85 on Prolific approval ratings, and 5) have no history of any diagnosed sleep, psychiatric, or neurological disorders. Of the eligible participants, 273 did not complete the follow-up experimental sessions. A total of 281 participants completed the study (*M*_*age*_ = 38.20, *SD*_*age*_ = 12.45). The majority of the sample identified as White (80.4% White, 9.6% Asian, 7.8% Black/African American, 0.7% American Indian/Native Alaskan, 0.4% Native Hawaiian/Pacific Islander, and 1.1% other; 7.5% Hispanic/Latino/Spanish). Approximately half of the sample reported their biological sex as female (53.4% female, 46.3% male, .4% not reported, and 0% intersex). Median annual household income was 70,000 U.S. dollars (range 2,500 to 335,00). Procedures were approved by the institutional review board at the University of Notre Dame. Participants gave written informed consent and received compensation through Prolific payments for their participation.

### Materials

#### Internalizing symptoms

The Mini-Mood and Anxiety Symptom Questionnaire (Mini-MASQ) is a short form of the Mood and Anxiety Symptom Questionnaire (MASQ) and provides a valid and reliable measure of internalizing symptoms (Casillas & Clark, 2000). The Mini-MASQ has 26 items and measures three dimensions of internalizing symptoms proposed by the Tripartite Model of Anxiety and Depression (Clark & Watson, 1991), including the common internalizing dimension “general distress” (8 items; e.g., “Felt like a failure”), anxiety-specific dimension “anxious arousal” (10 items; e.g., “Was short of breath”), and depression-specific dimension “anhedonia” (8 items; e.g., “Felt like nothing was very enjoyable”). See Table 1 for all items on the Mini-MASQ and their descriptive statistics in our sample. For each item, participants rated how much they “have felt or experienced things this way during the past week, including today” on a scale from 1 [not at all] to 5 [extremely].

**Table 1:** Common, depression-specific, and anxiety-specific items on the Mini-MASQ

Item	Wording/content	*M* (*SD*)
	*Common internalizing: general distress*	
C1	Felt tense or “high strung”	2.28 (1.12)
C2	Felt depressed	1.83 (1.04)
C3	Felt hopeless	1.48 (0.86)
C4	Felt keyed up, “on edge”	1.83 (1.05)
C5	Felt worthless	1.46 (0.95)
C6	Felt like a failure	1.54 (0.96)
C7	Felt uneasy	1.74 (0.98)
C8	Felt discouraged	1.77 (0.99)
	***Depression-specific: anhedonia***	
D1	Felt withdrawn from other people	1.85 (1.07)
D2	Felt like nothing was very enjoyable	1.63 (0.96)
D3*	Felt really happy	2.81 (1.03)
D4*	Felt like I had a lot to look forward to	3.05 (1.22)
D5*	Felt like I had a lot of interesting things to do	2.97 (1.15)
D6*	Felt really lively, “up”	3.45 (1.11)
D7*	Felt like I had a lot of energy	3.19 (1.07)
D8*	Felt like I was having a lot of fun	3.29 (1.08)
***Anxiety-specific: anxious arousal***		
A1	Was short of breath	1.32 (0.71)
A2	Felt dizzy or lightheaded	1.32 (0.67)
A3	Hands were cold or sweaty	1.38 (0.86)
A4	Hands were shaky	1.28 (0.73)
A5	Had trouble swallowing	1.11 (0.46)
A6	Had hot or cold spells	1.42 (0.83)
A7	Felt like I was choking	1.13 (0.53)
A8	Muscles twitched or trembled	1.29 (0.70)
A9	Was trembling or shaking	1.22 (0.65)
A10	Had a very dry mouth	1.38 (0.82)

C common internalizing factor, D depression-specific factor, A anxiety-specific factor

*Reverse-coded items and descriptive statistics

### Emotional memory trade-off task

**Fig. 1:**
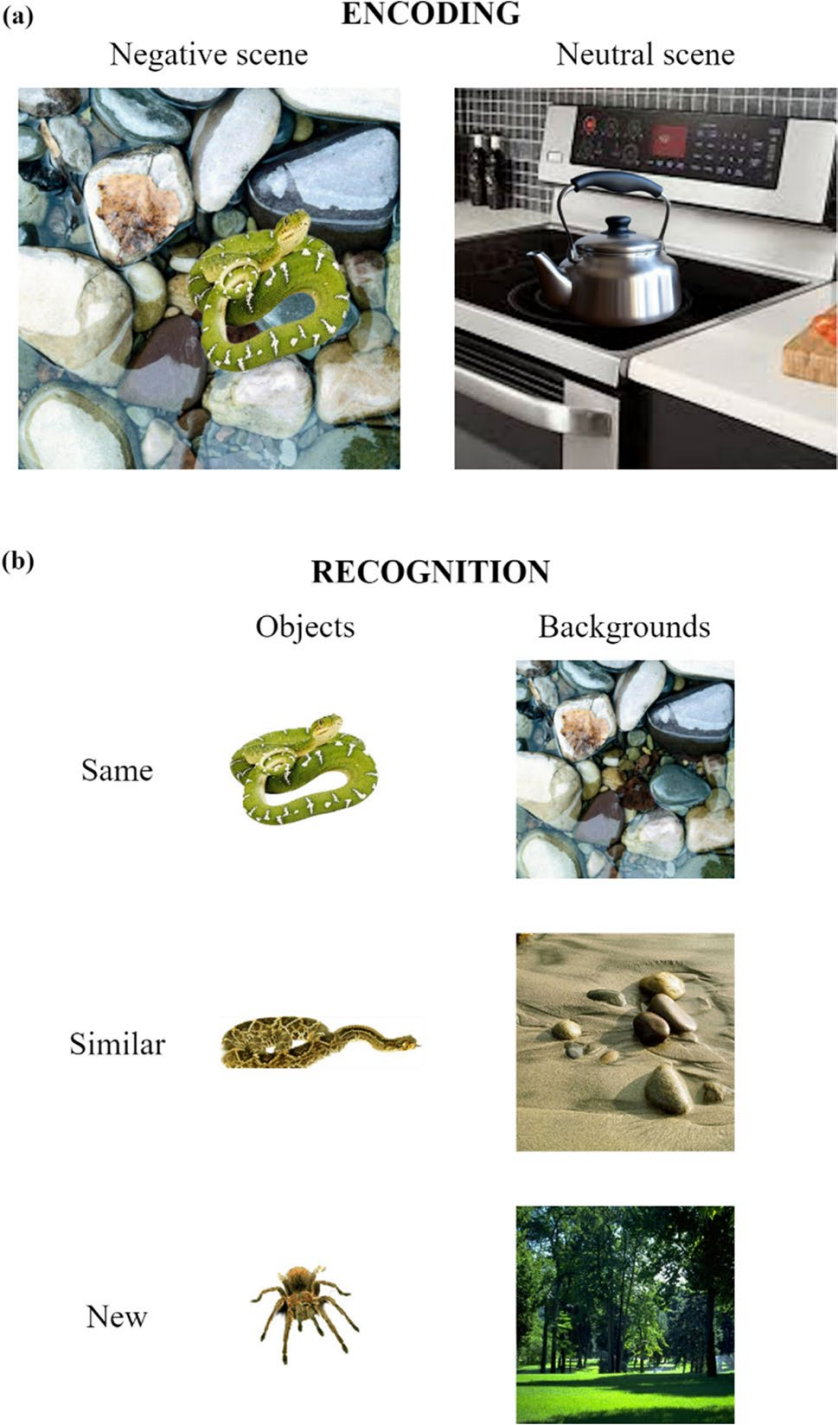
Stimuli during encoding and recognition. (**a**) Encoding stimuli. A negative scene might consist of a snake placed on wet pebbles, whereas a neutral scene might consist of a kettle placed on a kitchen stove. (**b**) Retrieval stimuli. A “same” item would be identical to components of scenes that they viewed during encoding. A “similar” item would share the same verbal label to a viewed scene component but differed in specific visual details. A “new” item would not have been seen during encoding. The presentation of "same" and "similar" versions of a particular item was counterbalanced among participants, ensuring that no participant encountered both versions during the retrieval test.

During incidental encoding, participants studied a series of 64 scenes in a random order, with each scene presented for 5 seconds. All scenes were composed of an object placed on a plausible neutral background. Half of the scenes portrayed a negatively arousing object (*negative scenes*), and the other half portrayed a neutral, nonarousing object (*neutral scenes*). Figure 1a demonstrates examples of negative and neutral scenes. After viewing each scene, participants rated two characteristics: valence and arousal. For valence, participants rated “how positive (pleasant) or negative (unpleasant) the scene is” on a scale from 1 [highly negative] to 7 [highly positive]. For arousal, participants rated “how calming/subduing or exciting/agitating you find the scene to be” on a scale from 1 [highly calming/subduing; makes you feel very relaxed, sleepy, etc.] to 7 [highly agitating or exciting; makes you feel highly awake, alert, aroused, etc.]. Participants were instructed to make these ratings based on their first impressions and not to overthink their responses (Denis et al., 2022; Niu et al., 2024). The average valence and arousal ratings of negative and neutral scenes were calculated. Our sample rated negative scenes as significantly more negative and more arousing than neutral scenes (Denis et al., 2022).

During recognition, memory performance was tested separately for objects and backgrounds. A total of 192 items were presented to participants; each item was presented for up to 10 seconds. The 192 items comprised three categories: 64 *same* items that participants had previously studied during encoding; 64 *similar* items that had the same verbal labels as those encountered during encoding but differed in specific visual details; and 64 *new* items that participants had never seen. Each category consisted of 32 objects (16 negative and 16 neutral) and 32 neutral backgrounds. For each item, participants indicated whether an object or a background was “same,” “similar,” or “new” compared with components of scenes that they viewed during encoding (Fig. 1b).

Hit rates were calculated as the proportion of trials participants responded “same” to a previously studied item, and false-alarm rates were calculated as the proportion of trials participants responded “same” to a new item. Recognition memory was calculated as the difference between hit and false-alarm rates. Trade-off memory was calculated as the difference in recognition memory between objects and the original backgrounds on which they were placed. Specifically, the negative memory trade-off effect is the difference in recognition memory between negative objects and their paired neutral backgrounds, and the neutral memory trade-off effect is the difference between neutral objects and their paired neutral backgrounds. The full memory trade-off effect is the difference between emotional and neutral memory trade-off effects.

### Procedure

Participants saw postings on Prolific titled “Emotional reactivity at different times of day,” which in turn directed them to complete an eligibility screening survey. Eligible participants were randomly assigned to one of the two delay conditions: a daytime wake condition, and a nighttime sleep condition. Participants in the daytime wake condition completed session 1 in the morning (7 to 11 a.m. local time), went about their daily routine except for napping, and performed session 2 in the evening on the same day (7 to 11 p.m. local time). Participants in the nighttime sleep condition performed session 1 in the evening (7 to 11 p.m. local time), were told to sleep how they normally would, and completed session 2 in the morning on the next day (7 to 11 a.m. local time). To ensure optimal data collection, we instructed participants to complete both experiment sessions by using a laptop or desktop computer with a compatible web browser, such as Google Chrome or Edge. Participants attempting the experiment on an incompatible device (mobile device, tablet, or using Internet Explorer) would receive an error message and be prompted to try again with a suitable setup.

During session 1, participants completed questionnaires assessing their subjective sleep and well-being and performed a modified brief 3-min version of the Psychomotor Vigilance Test (PVT-B) that measured subjective alertness (Basner et al., 2011). Participants then viewed 64 scenes one at a time that depicted a negative or neutral object placed on a neutral background. Participants were not told that there would be a later memory test but viewed these object-background associations and rated each scene for its valence and arousal (i.e., rated the full scenes with the objects and backgrounds together). Finally, participants responded to additional questionnaires to report their sleep and well-being, including the Mini-MASQ. Similarly, session 2 also started and ended with participants completing questionnaires that assessed their sleep and well-being. After the questionnaires, participants performed another 3-min Psychomotor Vigilance Test. Then, they were presented with 96 objects and 96 backgrounds separately, one at a time, and indicated whether each object or background was “same,” “similar,” or “new” compared with components of scenes that they viewed during the first session. Finally, they completed additional surveys on sleep and well-being. The scene viewing and memory tasks were programmed by using jsPsych (de Leeuw, 2015) and administered through Cognition.run (https://www.cognition.run). All questionnaires were built and distributed on Qualtrics (Qualtrics, Provo, UT).

### Analyses

The following procedures were performed by using Mplus (Muthén & Muthén, 2017). First, we conducted Confirmatory Factor Analysis (CFA) to determine whether bifactor S-1 models fit our data well. Good model fit criteria were: root-mean-square error of approximation (RMSEA) ≤ .06, comparative fit index (CFI) ≥ .97, and standardized root mean squared residual (SRMR) ≤ .08. Acceptable criteria were: RMSEA ≤ .08, CFI ≥ .95, and SRMR ≤ .10 (An et al., 2017; Cangur & Ercan, 2015). Second, we used Structural Equation Modeling (SEM) to examine common (i.e., general distress), depression-specific (i.e., anhedonia), and anxiety-specific (i.e., anxious arousal) internalizing symptoms as predictors for memory. We used full information maximum likelihood (FIML) to estimate missing data. The threshold for statistical significance was set to *p* < .05, two-tailed. The two-stage sharpened method (Benjamini et al., 2006) was used to control the false discovery rate (FDR) for individual parameters in SEM models. Our hypotheses and analytic plans were preregistered before data analysis.^1^ We report descriptive statistics and bivariate correlations of key study variables in Supplementary Materials Tables S1-S3 (https://doi.org/10.17605/OSF.IO/YJC4S).

#### Goal 1

**Fig. 2:**
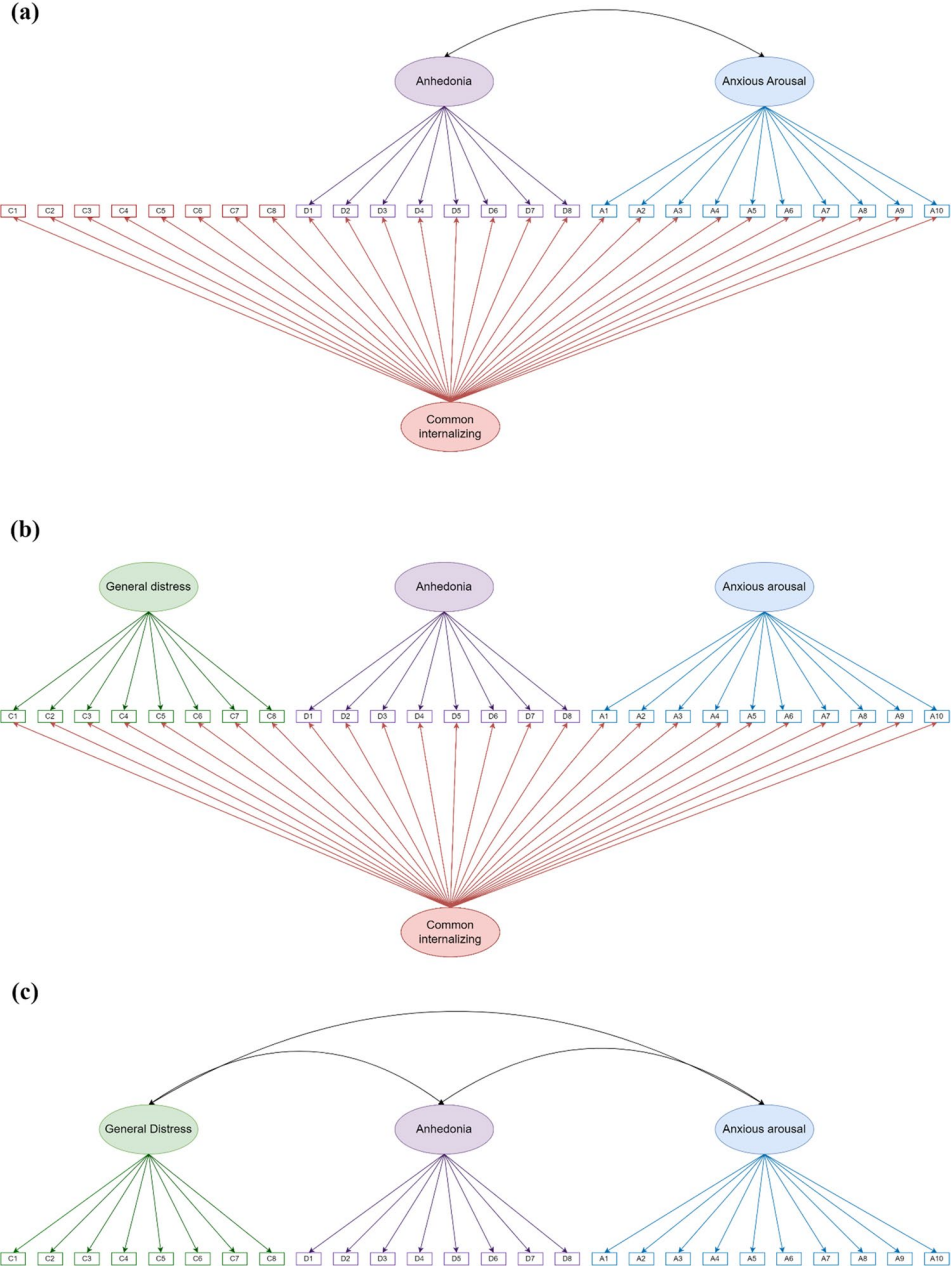
Confirmative factor analysis (CFA) model demonstration. (**a**) CFA for bifactor S-1 models. All items were loaded onto a common internalizing factor. Items from the anhedonia subscale were additionally loaded on a depression-specific anhedonia factor. Items from the anxious arousal subscale were additionally loaded on an anxiety-specific anxious arousal factor. Items from the general distress subscale were only loaded on a common internalizing factor and as a result represented the reference domain. Anhedonia and anxious arousal factors were allowed to correlate with each other but were both orthogonal to the common internalizing factor. (**b**) CFA for bifactor models. All items were loaded onto a common internalizing factor, and therefore there was no reference domain. Items from the general distress subscale were additionally loaded on a general distress factor. Items from the anhedonia subscale were additionally loaded on an anhedonia factor. Items from the anxious arousal subscale were additionally loaded on an anxious arousal factor. Common internalizing, general distress, anhedonia, and anxious arousal factors were all orthogonal to each other. (**c**) CFA for three-factor models. Items from the general distress subscale were loaded on a general distress factor. Items from the anhedonia subscale were loaded on an anhedonia factor. Items from the anxious arousal subscale were loaded on an anxious arousal factor. General distress, anhedonia, and anxious arousal factors were allowed to correlate with one another. C = common internalizing factor; D = depression-specific factor; A = anxiety-specific factor. *Reverse-coded items

For our first goal to disentangle common and specific dimensions of internalizing symptoms as proposed by the Tripartite Model of Anxiety and Depression (Clark & Watson, 1991), we ran CFA for bifactor S-1 models to load items on depression-specific anhedonia and anxiety-specific anxious arousal factors where general distress represented the common internalizing factor (Fig. 2a). Anhedonia and anxious arousal factors were allowed to correlate with each other but were both orthogonal to the common internalizing factor (Eid et al., 2017). We used chi-square (χ^2^) difference tests to compare models constraining anhedonia and anxious arousal factors to be orthogonal and models, allowing these two specific factors to freely correlate. Constraints on specific factors were freed when constraining them significantly hurt model fit statistics. For model comparisons, we performed two additional CFAs. These analyses examined a bifactor model that loaded items on common internalizing, general distress, anhedonia, and anxious arousal factors without a reference domain (Fig. 2b) and a three-factor model that load items on general distress, anhedonia, and anxious arousal factors as first-order factors where all factors were allowed to correlate (Fig. 2c).

In final selected CFA models, we conducted multigroup CFAs to test measurement invariance between delay conditions. In 258 participants with non-missingness on the MINI-MASQ, we determined whether factor loadings and intercepts were similar between the daytime wake condition (*n* = 129) and nighttime sleep condition (*n* = 129). Considering the important roles of sex and age in internalizing symptoms (Luppa et al., 2012; Niu et al., 2021), we also performed multigroup CFAs to compare females (*n* = 134) with males (*n* = 123) and to compare young adults (18–35 years, *n* = 106) with middle-aged adults (36–59 years, *n* = 152). Model fit indices were examined for 1) configural invariance where both factor loadings and intercepts were freed to evaluate whether the overall factor structure fit well across groups, 2) metric invariance where factor loadings were constrained and intercepts were freed to test whether factor loadings were equivalent across groups, and 3) scalar invariance where both factor loadings and intercepts were constrained to additionally compare intercepts across groups (Lee, 2018). We used the recommended guidelines of measurement invariance for sample sizes <300: ΔCFI < .010 and ΔRMSEA < .015 (Chen, 2007; Cheung & Rensvold, 2002).

#### Goal 2

For our second goal to evaluate the effects of common and specific dimensions of internalizing symptoms on emotional memory consolidation, we examined whether general distress, anhedonia, and anxious arousal were significant predictors for outcomes in six separate SEMs. Each model tested multiple outcome variables simultaneously. Model 1 examined four outcomes: valence and arousal ratings for negative scenes at encoding, and valence and arousal ratings for neutral scenes at encoding. Model 2 examined two outcomes: the difference in valence ratings between neutral and negative scenes at encoding (i.e., subtracting valence for negative scenes from that for neutral scenes as lower valence ratings represent more negative), and the difference in arousal ratings between negative and neutral scenes at encoding (i.e., subtracting arousal for neutral scenes from that for negative scenes as lower arousal ratings indicate less arousing). Model 3 examined four outcomes: recognition memory for negative objects and their associated neutral backgrounds, as well as neutral objects and their associated neutral backgrounds. Model 4 examined two outcomes: the difference in recognition memory between negative objects and neutral objects, and the difference in recognition memory between backgrounds of negative scenes and background of neutral scenes. Model 5 examined two outcomes: the negative memory trade-off effects and the neutral memory trade-off effects. Model 6 examined one outcome which was the full memory trade-off effect. Additionally, we performed Wald tests to compare effect sizes of general distress, anhedonia, and anxious arousal on each of the outcome variables.

#### Goal 3

For our third goal to determine whether common and specific dimensions of internalizing symptoms were more associated with emotional memory consolidation after post-learning sleep compared to wakefulness, we conducted multigroup SEM analyses to compare the daytime wake (*n* = 129) and nighttime sleep conditions (*n* = 129) in six separate SEMs. Each of the six multigroup SEMs tested the same sets of outcomes as outlined in Goal 2, with each model testing multiple outcomes simultaneously. Finally, we performed Wald tests to determine whether any effect sizes differed significantly between the two delay conditions.

#### Power analysis

The power of this study was assessed based on Monte Carlo simulations with 1,000 iterations using MPlus (Muthén & Muthén, 2017) and a sample size of 258 participants with non-missingness on MINI-MASQ data. The percentage of significant coefficients were used to estimate the power for the effects of common and specific internalizing symptom dimensions on emotional memory in SEMs. We estimated factor loadings based on CFA results in the current study by using the bifactor S-1 model that extracted general distress, anhedonia, and anxious arousal factors from the MINI-MASQ and allowed anhedonia and anxious arousal to correlate. To standardize factor loadings, we fixed variances of measurement errors for both manifest variables and latent factors to one and fixed factor means to zero. As demonstrated in Table S9, 91% (*n* = 41) of the 45 factor loadings demonstrated sufficient power (>.80). Because we are the first to investigate the effects of common and specific internalizing symptoms on emotional memory, we performed a sensitivity analysis to determine the minimum effect sizes for which we had adequate power in SEM analyses given the sample size of 258 participants. The estimated parameters for the percentage of significant coefficients to approach .80 was 0.20 for general distress, 0.25 for anhedonia, and 0.28 for anxious arousal.

## Results

### Confirmative factor analyses

Table 1 shows descriptive statistics for all items measuring internalizing symptoms. Table S4 displays model fit statistics for four different CFA models: 1) the conventional bifactor model; 2) the bifactor S-1 model allowing correlations between specific factors; 3) the bifactor S-1 model constraining specific factors to be orthogonal; and 4) the three-factor model. See Table S5 to Table S8 for full CFA results.

**Fig. 3:**
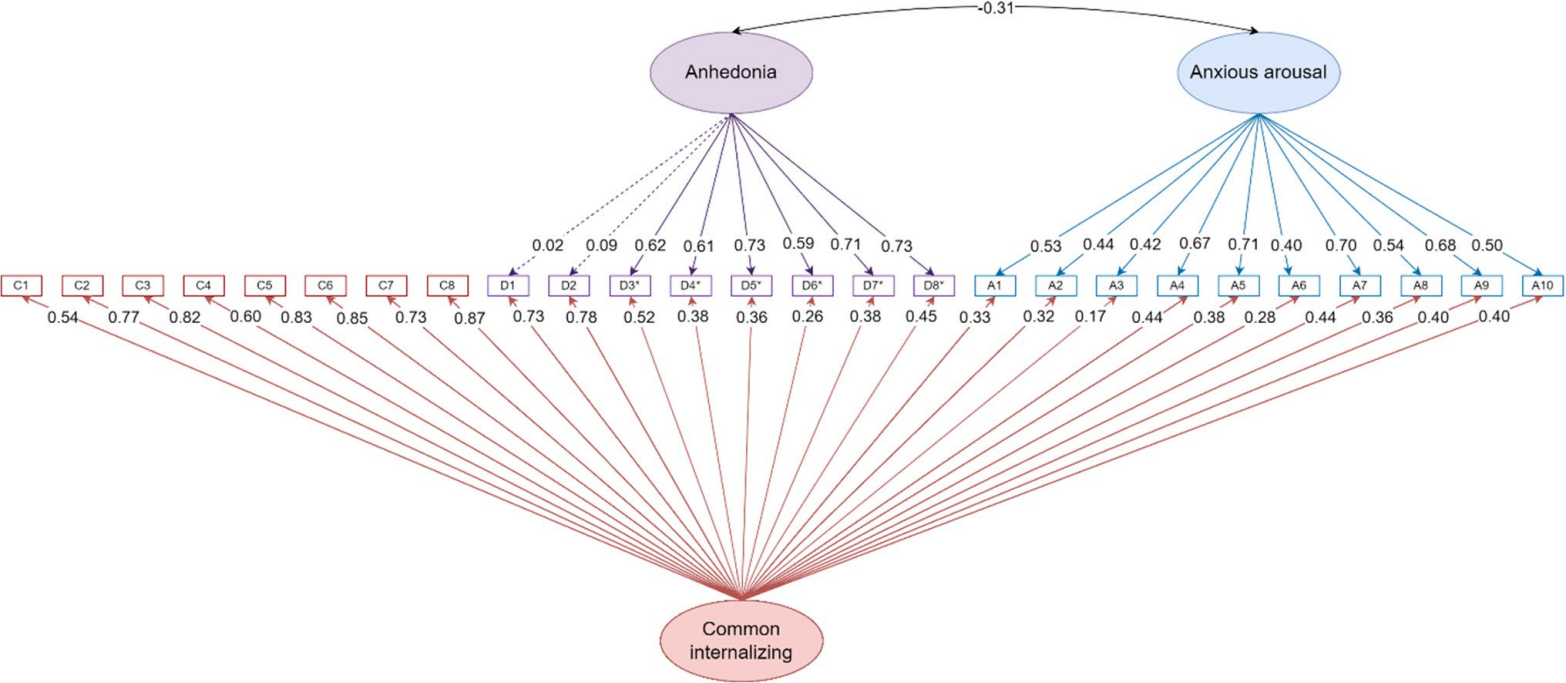
Confirmative factor analysis (CFA) of the final selected bifactor S-1 model

The CFA model with the best fit was the conventional bifactor model (RMSEA = .06, CFI = .91, SRMR = .07, AIC = 13582.71, BIC = 13952.22). However, because of negative factor loadings on the general distress factor (Table S7) and the ambiguity surrounding the common factor in the bifactor model, we selected the next best-fitting model: the bifactor S-1 model allowing anhedonia and anxious arousal to freely correlate (Fig. 3). The correlation between these two specific factors is −0.31, which was significantly different from 0 (*p* = .001). This bifactor S-1 model demonstrated good to acceptable model fit statistics (RMSEA = .08, CFI = .86, SRMR = .06, AIC = 13790.80, BIC = 14135.43). It showed better model fits than the bifactor S-1 model constraining specific factors to be orthogonal (RMSEA = .08, CFI = .85, SRMR = .07, AIC = 13807.30, BIC = 14148.38) and the three-factor model ((RMSEA = .09, CFI = .79, SRMR = .12, AIC = 14060.53, BIC = 14348.32). In the final selected bifactor S-1 model, most items significantly loaded onto the general distress, anhedonia, and anxious arousal factors (*ps* < .03, Fig. 3; Table S5), except for D1 (“Felt withdrawn from other people”) and D2 (“Felt like nothing was very enjoyable”) on the anhedonia factor. The bifactor S-1 achieved scalar and metric measurement invariance for delay conditions and age and achieved scalar measurement invariance for sex (Table S10).

This bifactor S-1 model used general distress as a reference domain for the common internalizing factor and allowed depression-specific anhedonia and anxiety-specific anxious arousal factors to freely correlate. Straight arrows indicate standardized factor loadings, and curved arrows indicate correlated item residuals. Solid lines indicate significant effects, and dashed lines indicate nonsignificant effects. C = common internalizing factor; D = depression-specific factor; A = anxiety-specific factor. *Reverse-coded items.

### Recognition memory

Table 2 summarizes standardized SEM results examining general distress, anhedonia, and anxious arousal as predictors for recognition memory of negative objects and associated backgrounds, as well as neutral objects and associated backgrounds, in the total sample, and separately for the daytime wake condition and nighttime sleep condition. See *Supplementary Materials* Tables S15-S18 for full model results and Table S32 for Wald’s comparisons of effect sizes.

**Table 2:** Standardized SEM results of internalizing symptoms measured by the bifactor S-1 model predicting gist and specific recognition memory in the daytime wake condition, nighttime sleep condition, and total sample

		General distress	Anhedonia	Anxious arousal
**Object memory**				
**Negative scenes**	Wake	-0.01	-0.04	-0.34^***^
	Sleep	0.06	-0.09	-0.22
	Total	0.01	-0.06	-0.30^***^
**Neutral scenes**	Wake	-0.11	0.03	-0.26^**^
	Sleep	0.12	-0.11	-0.15
	Total	-0.01	-0.06	-0.24^**^
**Object difference**	Wake	0.13	-0.09	-0.14
	Sleep	-0.06	0.02	-0.12
	Total	0.03	0.00	-0.11
**Background memory**				
**Negative scenes**	Wake	0.02	-0.06	-0.30^***^
	Sleep	0.12	0.07	-0.20⁺
	Total	0.06	0.00	-0.26^***^
**Neutral scenes**	Wake	0.02	-0.19⁺	-0.30^***^
	Sleep	0.11	0.05	-0.16
	Total	0.05	-0.06	-0.24^**^
**Background difference**	Wake	0.00	0.19	0.08
	Sleep	0.00	0.02	-0.02
	Total	0.01	0.08	0.04

Object difference, the difference in object memory between negative and neutral scenes, where a positive score indicates better memory for negative objects compared to neutral objects. Background difference, the difference in background memory between negative and neutral scenes, where a positive score indicates better memory for backgrounds of negative scenes compared with backgrounds of neutral scenes. Significant effects after the two-stage controlling procedure for the false-discovery rate were in bold: *<.05; **<.01; ***<.001. ⁺Significant effects only before adjusting for multiple comparisons

#### Full sample

Higher anxious arousal predicted worse memory for all scene components, including negative objects (*β* = −0.30, *p* < .001) and associated backgrounds (*β* = −0.26, *p* < .001), as well as neutral objects (*β* = −0.24, *p* = .002) and associated backgrounds (*β* = −0.24, *p* = .002). General distress and anhedonia were not associated with memory for any scene components (*ps* > .6; Table S15). None of the internalizing symptoms were associated with differences in object or background memory between negative and neutral scenes (*ps* > .9; Table S17). Full SEM results controlling for sex, age, alertness, and encoding ratings of valence and arousal mostly followed the same pattern and are reported in Tables S36-S53.

#### Delay conditions

Anxious arousal significantly predicted worse memory across all scene components only when retention intervals spanned daytime wakefulness, including objects (*β* = −0.34, *p* < .001) and backgrounds (*β* = −0.30, *p* < .001) from negative scenes, as well as objects (*β* = −0.26, *p* = .005) and backgrounds (*β* = −0.30, *p* < .001) from neutral scenes. Although these effects were not observed in the nighttime sleep condition (*ps* > .3; Table S16), effect sizes between the two delay conditions did not differ significantly (*ps* > .2; Table S32). The effects of general distress on memory performance for neutral objects differed significantly between the daytime wake and nighttime sleep conditions (^*2*^(1) = 4.95, *p* = .026). Although not significantly, increased general distress was linked to worse memory for neutral objects after a day spent awake (*β* = −0.11, *p* = .298), but better memory after a night spent asleep (*β* = 0.12, *p* = .298). Full SEM results excluding 30 participants who reported napping during the retention interval largely followed the same pattern, and are reported in Tables S54-S59.

#### Memory trade-off effects

There were no significant effects of general distress, anhedonia, or anxious arousal on negative, neutral, or full memory trade-off effects in the total sample or separately for the daytime wake and nighttime sleep conditions (*ps* > .9). See *Supplementary Materials* Tables S19-S22 for full model results and Table S33 for Wald’s comparisons of effect sizes.

#### Valence and arousal ratings

Table 3 summarizes standardized SEM results examining general distress, anhedonia, and anxious arousal as predictors for valence and arousal ratings in the total sample, and separately for the daytime wake condition and nighttime sleep condition. See *Supplementary Materials* Tables S11-14 for full model results and Table S31 for Wald’s comparisons of effect sizes.

**Table 3:** Standardized SEM results of internalizing symptoms measured by the bifactor S-1 model predicting valence and arousal ratings in the daytime wake condition, nighttime sleep condition, and total sample

		General distress	Anhedonia	Anxious arousal
**Valence ratings**				
**Negative scenes**^※^	Wake	-0.04	0.07	0.27⁺
	Sleep	-0.01	0.12	0.19
	Total	-0.03	0.10	0.24⁺
**Neutral scenes**^※^	Wake	-0.21^*^	-0.41^***^	-0.24^***^
	Sleep	0.20⁺	-0.28^*^	0.13
	Total	-0.04	-0.35^***^	-0.13
**Valence Difference**	Wake	-0.11	-0.33^**^	-0.37^***^
	Sleep	0.16	-0.32^**^	-0.07
	Total	-0.01	-0.33^***^	-0.28^**^
**Arousal ratings**				
**Negative scenes**	Wake	-0.06	-0.06	-0.26
	Sleep	0.08	-0.17	-0.05
	Total	-0.01	-0.12	-0.19
**Neutral scenes**	Wake	0.10	0.08	0.27^**^
	Sleep	0.04	0.37^***^	0.35^*^
	Total	0.06	0.22^**^	0.33^***^
**Arousal Difference**	Wake	-0.12	-0.10	-0.41^***^
	Sleep	0.02	-0.39^***^	-0.30^*^
	Total	-0.06	-0.25^***^	-0.39^***^

^※^As valence is rated on a scale from 1 [very negative] to 7 [very positive], a positive score indicates more positive, less negative ratings. Valence difference, valence ratings for neutral scenes minus that for negative scenes, where a positive score indicates that negative scenes are rated as more negative than neutral scenes. Arousal difference, the difference in arousal ratings between negative and neutral scenes, where a positive score indicates that negative scenes are rated as more arousing than neutral scenes. Significant effects after the two-stage controlling procedure for the false-discovery rate are in bold: *<.05; **<.01; ***<.001. ⁺Significant effects only before adjusting for multiple comparisons

### Full sample

Both anhedonia (*β* = −0.33, *p* < .001) and anxious arousal (*β* = −0.28, *p* = .002) were associated with rating negative and neutral scenes as similarly negative.^2^ However, this pattern emerged differently: anhedonia was significantly related to rating neutral scenes as more negatively valenced (*β* = −0.35, *p* < .001), whereas anxious arousal was linked to perceiving negative scenes as less negative (*β* = 0.24, *p* = .052)^3^. In addition, higher levels of both anhedonia (*β* = 0.22, *p* = .009) and anxious arousal (*β* = 0.33, *p* < .001) were associated with rating neutral scenes as more arousing. Correspondingly, anhedonia (*β* = −0.25, *p* = .001) and anxious arousal (*β* = −0.39, *p* < .001) both predicted rating neutral scenes as similarly arousing as negative scenes. Wald’s comparisons of effect sizes showed that anhedonia and anxious arousal overall had similar effects on valence and arousal ratings (Table S31). Notably, both anhedonia and anxious arousal predicted rating neutral scenes as similarly negative and arousing as negative scenes, and these less differentiated valence and arousal ratings correlated with worse memory across all scene components (Table S2). There were no significant effects of general distress on either valence or arousal ratings (*ps* > .7; Tables S11 and S13).

### Delay conditions

To further investigate the effects of internalizing symptoms on valence and arousal ratings, we examined their respective estimates in the daytime wake (morning) and nighttime sleep (evening) conditions. General distress demonstrated significantly different effects on valence ratings of neutral scenes in the morning and evening (${\chi}^{2}$(1) = 8.46, *p* = .004): higher general distress was associated with rating neutral scenes more negatively in the morning (*β* = −0.21, *p* = .041), but a pattern of rating neutral scenes less negatively in the evening (*β* = 0.20, *p* = .070). General distress did not predict arousal ratings in either condition (*ps* > .5; Tables S12 and S14).

Higher anhedonia predicted rating neutral scenes as more negative both in the morning (*β* = −0.41, *p* < .001) and evening (*β* = −0.28, *p* = .014), and rating negative and neutral scenes as similarly negative both in the morning (*β* = −0.33, *p* = .001) and evening (*β* = −0.32, *p* = .001). Wald’s comparisons of effect sizes showed that anhedonia had similar effects on valence ratings in morning and evening (Table S31). The effects of anhedonia on arousal ratings for neutral scenes, however, were significantly different between morning and evening (${\chi}^{2}$(1) = 4.26, *p* = .039). Specifically, anhedonia was linked to rating neutral scenes as more arousing (*β* = 0.37, *p* < .001) and similarly arousing as negative scenes (*β* = −0.39, *p* < .001) only in the evening but not in the morning (Table S14).

The effects of anxious arousal on valence ratings for neutral scenes were significantly different between morning and evening (${\chi}^{2}$(1)=12.84, *p* < .001): higher anxious arousal was linked to rating neutral scenes as more negative only in the morning (*β* = −0.24, *p *< .001) but not evening (Table S14). Relatedly, anxious arousal predicted rating neutral and negative scenes as similarly valenced only in the morning (*β* = −0.37, *p* < .001) but not evening (Table S14), and this comparison was also significant (${\chi}^{2}$(1) = 5.46, *p* = .019). The effects of anxious arousal on arousal ratings were similar in morning and evening (Table S31). Higher anxious arousal was related to rating neutral scenes as more arousing both in the morning (*β* = 0.27,* p* = .009) and evening (*β* = 0.35, *p* = .021), as well as rating negative and neutral scenes as similarly arousing both in the morning (*β* = −0.41, *p* < .001) and evening (*β* = −0.30, *p* = .048).

## Discussion

The goal of the current study was to investigate the effects of common, depression-specific, and anxiety-specific internalizing symptoms on the consolidation of negative and neutral episodic memories over a 12-hour delay period. Our findings do not support the idea that internalizing symptoms are associated with an increased emotional memory bias (i.e., better memory for negative compared to neutral information). Instead, anxiety-specific internalizing symptoms (i.e., anxious arousal) impaired overall memory performance, regardless of whether the information was emotionally negative or neutral. Notably, however, these anxiety-specific memory impairments were only significant when the retention interval spanned wakefulness; the same was not the case when the retention interval included sleep.

None of the internalizing symptoms predicted recognizing more negative objects than neutral objects from separate scenes or associated neutral backgrounds from the same scenes. These results are inconsistent with previous findings that suggest that depression and anxiety both individually (Everaert et al., 2014; Fattahi Asl et al., 2015; Greden, 1982; Hakamata et al., 2022; Miles et al., 2004) and collectively (Coles et al., 2007; Dowens & Calvo, 2003; Gilboa-Schechtman et al., 2002; LeMoult & Joormann, 2012) contribute to enhanced memory for negative information compared with neutral information. There are several possible explanations for these null effects. First, many studies that have reported an association between internalizing symptoms and emotional memory bias were conducted among clinically depressed and/or anxious individuals (Coles et al., 2007; Dalgleish et al., 2003; Fattahi Asl et al., 2015; Gilboa-Schechtman et al., 2002; Howe & Malone, 2011; MacLeod et al., 1997). Given that our study examined generally healthy adults from a community sample, it is unlikely to be sensitive enough to capture characteristics related to information and memory processing that are only present in those with clinically severe symptoms of depression and anxiety.

Second, we hypothesized that internalizing symptoms would predict enhanced negative episodic memories owing to their associations with prolonged negative emotional processing during sleep (Niu et al., 2023; Niu & Snyder, 2022; Taylor & Snyder, 2021). However, because of deficits in executive functioning and motivation, individuals with more severe depression-related and anxiety-related internalizing symptoms also experience general memory declines for both negative and neutral information (Grahek et al., 2019; Snyder & Hankin, 2019). These memory-related deficits may grow exponentially over the 12-hr delay periods, and eventually overshadow the negative memory bias (Dillon & Pizzagalli, 2018). Moreover, most previous studies employed simple word-list stimuli (Coles et al., 2007; Dalgleish et al., 2003; Dowens & Calvo, 2003; Everaert et al., 2014; Fattahi Asl et al., 2015; Hakamata et al., 2022; Howe & Malone, 2011), whereas our encoding materials included complex image stimuli depicting scenes with objects placed on backgrounds. These stimuli are more cognitively demanding and susceptible to accelerated forgetfulness associated with internalizing symptoms (Hammar & Årdal, 2013; Schweizer et al., 2018; Wang et al., 2022; Wong et al., 2000).

Another possible explanation for the null effects of internalizing symptoms on negative memory bias is that individuals with higher internalizing symptoms do not differentiate between negative and neutral scenes more than those with no or fewer symptoms. In other words, compared with those with lower internalizing symptoms, individuals with more severe symptoms may not “tag” negative information as more salient than neutral information or allocate additional cognitive resources when encoding negative information (Everaert et al., 2014; Greene et al., 2010; Mathews & MacLeod, 2005; McIntyre & Roozendaal, 2007). As a result, although emotionally negative experiences are typically better remembered than neutral experiences (Brown & Kulik, 1977; Holland & Kensinger, 2010; Kensinger, 2009), this emotional memory bias is not further enhanced by internalizing symptoms.

In line with this explanation, we found that individuals with more severe internalizing symptoms perceived neutral information as more negative and arousing but did not rate negative information as more negative or arousing compared with those with lower symptoms. These findings only partially support past research that internalizing symptoms are linked to perceiving all information, regardless of whether it is negative or neutral, as more negative and arousing (Dieleman et al., 2010; Eden et al., 2015; Teismann et al., 2020). One explanation is that the negative stimuli in our study were not intentionally chosen to evoke emotional states related to depression or anxiety. Previous studies often have used more mood-congruent stimuli pertinent to depression and anxiety, such as sad facial expressions, crying sounds, or words related to anxiety (Beevers et al., 2019; Kanske & Kotz, 2012; Young et al., 2020). In contrast, most negative stimuli in our study are designed to universally evoke negative and arousing responses (e.g., images of vicious looking snake, weapon, car accident), potentially reaching functional ceiling levels of negative valence (*M* = 2.68 on a 1–7 scale, with 1 being the most negative) and arousal (*M* = 5.33 on a 1–7 scale, with 7 being the most arousing), even among participants with less severe internalizing symptoms. Previous studies indicate that depressed and anxious individuals may not exhibit a stronger bias toward universally negative information compared with those with lower symptoms (Everaert et al., 2014; Mathews & MacLeod, 2005), but instead they tend to regard ambiguous stimuli as more negative or threatening (Cohen, 2013; Lawson & MacLeod, 1999). It also is possible that negative scenes produced a carry-over effect, influencing the ratings of subsequent neutral scenes to be perceived as more negative and arousing. Individuals with higher depression and anxiety may be especially sensitive to such carry-over effects (Girondini et al., 2023; Hegarty et al., 2016).

We found that compared to individuals with lower internalizing symptoms, those with greater general distress, anhedonia, and anxious arousal all rated neutral scenes as more negative during morning hours (7–11 a.m.). This is potentially due to the typical circadian rhythm of cortisol awakening responses (i.e., peak cortisol levels after waking up; Chida & Steptoe, 2009; O’Byrne et al., 2021). As internalizing symptoms often co-occur with shorter sleep duration and worse sleep quality, individuals with more severe depression or anxiety symptoms tend to experience higher cortisol awakening responses, contributing to an increased negativity bias in the early morning hours. As cortisol levels gradually decline throughout the day, the negativity bias also decreases in the late evening or late night (Kanske & Kotz, 2012; O’Byrne et al., 2021; Young et al., 2020). This may relate to why individuals with higher general distress and anxious arousal did not rate neutral scenes more negatively in the evening compared with those with no or fewer symptoms (7–11 p.m.). Interestingly, anhedonia predicted increased negative interpretation bias of neutral information both in the morning and evening. One reason for this is because depression symptoms are associated with disruptions in the diurnal cortisol rhythm, characterized by impaired recovery following cortisol awakening responses and elevated evening cortisol levels (Adam et al., 2017; Burke et al., 2005; Dieleman et al., 2010; Vreeburg et al., 2009).

The current study replicated previously reported effects of anxiety on general memory impairments but did not replicate depression-related memory deficits (Dowens & Calvo, 2003; Hakamata et al., 2022; Howe & Malone, 2011; Kizilbash et al., 2002). More specifically, we found that anxiety-specific anxious arousal, but not general distress or anhedonia, impaired recognition memory across all scene components. Because past research did not properly separate the unique and shared contribution of depression and anxiety through bifactor modeling (Dowens & Calvo, 2003; Hakamata et al., 2022; Howe & Malone, 2011; Kizilbash et al., 2002), it is possible that memory deficits associated with depressive disorders or comorbid depressive and anxiety disorders may simply arise from the specific effects of anxious arousal. Notably, our findings successfully replicated a previous study that used the Tripartite Model of Anxiety and Depression (Clark & Watson, 1991) and found that only symptoms related to anxiety predicted poorer prospective memory performance (Bowman et al., 2019).

The different effects of anxiety and depression on general memory likely involve a complex interplay of cognitive, neurobiological, and psychological factors. For example, while the release of arousal-related neuromodulators (e.g., norepinephrine) is an adaptive response to acute stressors, anxiety is characterized by a chronic state of excessive anxious arousal (Morilak et al., 2005; Vismara et al., 2020). Individuals with high anxious arousal may constantly engage in maladaptive avoidance strategies for a temporary relief, potentially disrupting information processing and encoding (López-Moraga et al., 2022; Murty et al., 2011). These interferences could ultimately lead to memory deficits even in neutral or low-stress situations (López-Moraga et al., 2022; Murty et al., 2011). In contrast, depression-specific anhedonia, although characterized by reduced motivation and active engagement, may spare basic cognitive processes (Boehme et al., 2020; Thompson et al., 2010; Watson et al., 2020). The incidental nature of our encoding task, where participants passively viewed scenes without explicit instructions to remember stimuli, possibly captures passive sensory encoding, a process relatively unaffected in anhedonia, as opposed to processes linked to motivation and active engagement (Boehme et al., 2020; Thompson et al., 2010; Watson et al., 2020).

Consistent with previous studies that sleep mitigates memory deficits related to anxiety disorders (Davidson et al., 2021; Snyder & Hankin, 2019; Tsirimokos et al., 2022), we only observed anxiety-specific memory impairments when retention intervals spanned wakefulness but not sleep. A long-standing theory proposes that sleep protects against memory loss from daytime-related interference (Jenkins & Dallenbach, 1924). Particularly for individuals with severe anxiety symptoms, whose intrusive thoughts and feelings spread to a plurality of concerns during wakefulness, post-learning sleep may provide robust passive protection against daytime interferences (Casillas & Clark, 2000), especially during SWS where slow oscillatory activities may offer ameliorating benefits on anxious thoughts and cognitive disruptions (Ben Simon et al., 2020; Chellappa & Aeschbach, 2022). More recent literature suggests that sleep also plays a more active role in memory consolidation, facilitating the integration and redistribution of newly encoded information from the hippocampus to the neocortex for long-term storage (Ellenbogen et al., 2006; Klinzing et al., 2019). Given that individuals with high anxiety symptoms tend to struggle with allocating adequate attentional resources during encoding, active consolidation processes during sleep could help to stabilize weakly encoded information and mitigate anxiety-related learning impairments (LeMoult & Joormann, 2012; Snyder & Hankin, 2019; Tsirimokos et al., 2022).

Another project that used the same dataset found that sleep selectively benefited memories for the negative central event at the cost of peripheral neutral information in the background (Denis et al., 2022). Contrary to our hypotheses, however, this sleep-related enhancement in the emotional memory trade-off effect was not more robust among individuals reporting more severe internalizing symptoms. Our findings are consistent with a previous study reporting that sleep does not enhance emotional memory bias related to depression symptoms (Harrington, Nedberge et al., 2018b). However, it is still likely that specific sleep-related characteristics, such as prolonged REM sleep, are involved in the selective consolidation of emotional memories (Bennion, Payne et al., 2015b; Dudai et al., 2015; Klinzing et al., 2019). Previous literature indicates that only when post-learning retention intervals are rich in REM sleep would individuals with higher internalizing symptoms predict emotional memory bias (Harrington et al., 2017, Harrington, Johnson et al., 2018a). Increased measures of REM sleep markers are frequently identified as important clinical markers for depressive disorders (Palagini et al., 2013; Pillai et al., 2011; Riemann et al., 2001) and might not be as evident in our generally healthy community sample. As a result, negative memory bias might only emerge with a broader range of internalizing symptom severity and/or REM sleep dysregulation. Future studies will need to examine clinical samples experiencing depressive and/or anxiety disorders to directly test this hypothesis.

## Limitations and future directions

The current study has several limitations. First, although our sample was recruited from various communities across the United States and reflects a broader portion of society compared with typical college-student samples, it was still limited to healthy participants with the majority being White. In particular, our sample reported a restricted range of internalizing symptoms, particularly for anxiety-specific symptoms, although we nevertheless observed robust anxiety-related memory impairments. Regardless, this could still hinder our ability to generalize our findings to populations with clinically severe symptoms of depression and anxiety. More studies are needed to recruit less homogenous samples in terms of race, ethnicity, and severity of internalizing symptoms. Second, compared with overnight laboratory studies that collect whole nights of sleep EEG, our online experiment exerted lower degrees of experimental control and was unable to determine precise sleeping times or establish whether certain sleep stages are more specialized in the selection consolidation of emotional memories. Past research has found associations between REM sleep and emotional memory consolidation, as well as SWS and neutral memory consolidation, although findings are mixed (Groch et al., 2013, 2015; Harrington, Johnson et al., 2018a, Harrington, Nedberge et al., 2018b; Sopp et al., 2017; Wagner et al., 2001). More laboratory studies are needed to assess whether these associations are different among individuals experiencing a varying degree of internalizing symptoms.

Next, it is likely that differences between the daytime wake and nighttime sleep conditions do not arise from sleep effects alone but from time-of-day effects as well. Better performance in the morning could be due to sleep participants being well-rested and alert at retrieval, whereas poorer performance of wake participants in the evening could reflect accumulated fatigue and decreased alertness throughout the day (Jankowski & Zajenkowski, 2016; Wilks et al., 2021; Yaremenko et al., 2021). However, it is important to note that time-of-day effects might negatively impact the sleep group during encoding, which is generally considered more susceptible to reduced alertness compared to retrieval processes (Craik et al., 2018; Naveh-Benjamin et al., 2000). Still, circadian rhythms, time-of-day optimality, and diurnal variations related to internalizing psychopathology could all be influencing memory-related processes (Baran et al., 2012; Cunningham et al., 2014; Rusting & Larsen, 1998). Future research should employ more controlled designs to disentangle the effects of sleep from time-of-day influences on memory performance (Cunningham et al., 2022). For example, studies could include additional control groups that complete the encoding session in the evening (and/or morning) with an immediate memory test, as we have done in previous studies (Bennion, Mickley Steinmetz et al., 2015a; Payne & Kensinger, 2011). In addition, designs manipulating sleep, such as nocturnal sleep deprivation and daytime napping designs (Payne & Kensinger, 2011) could help to isolate the specific contribution of sleep and circadian effects.

Finally, the emotional memory trade-off task did not include an immediate memory task that is less sensitive to cognitive deficits commonly associated with internalizing symptoms (Grahek et al., 2019; Snyder & Hankin, 2019). Future research would benefit from including both immediate and delayed memory tasks to better separate negativity bias during encoding-related and consolidation-related processes. It also is important to note that our study period (March 2021) overlapped with the Covid-19 pandemic during which participants might have been facing various psychological, financial, and health-related stressors. It is important to acknowledge that the pandemic could have influenced the relationship between sleep, internalizing symptoms, and cognitive processes (Niu & Snyder, 2022).

## Conclusions

This is one of the first studies to attempt to disentangle common and specific dimensions of internalizing symptoms using bifactor S-1 modeling and examine these latent dimensions as predictors for emotional and neutral episodic memories. We provide well-powered evidence that anxiety-specific internalizing symptoms, rather than common or depression-specific ones, are associated with general memory impairments. Importantly, our findings highlight the protective role of post-learning sleep against memory deficits linked to anxiety symptoms. These findings suggest that interventions targeting sleep-related characteristics, such as improving sleep quality and increasing sleep duration, might help to mitigate learning-related impairments among individuals with higher anxiety symptoms.

## Supplementary Information

Below is the link to the electronic supplementary material.

Supplementary file1 (PDF 354 KB)

## Data Availability

Supplementary Materials, preregistration, data files and Mplus syntax & outputs are available at https://doi.org/10.17605/OSF.IO/YJC4S .

## Footnotes

1 Departures from preregistration.

- To better capture the memory impairments associated with internalizing symptoms, we reported a more stringent, specific recognition memory where a hit was scored as responding only “same” to an old item, as opposed to gist memory where a hit was scored as responding “same” or “similar” to an old item (Denis et al., 2022). Results for gist memory mostly followed the same patterns as specific memory and are reported in Supplementary Materials.
- To increase the statistical power of bifactor S-1 models, we did not allow any individual MASQ items to correlate.
- We reported SEM results without controlling for sex, age, and alertness as these variables were not part of our main research interests. Model results with sex, age, and alertness as covariables mostly followed the same pattern and are reported in *Supplementary Materials* Table S36 to Table S45.

2 The outcome here was subtracting valence ratings of negative scenes from valence ratings of neutral scenes. A negative parameter indicates differentiating negative scenes from neutral scenes to a lesser extent.

3 Participant rated the valence of each scene on a scale from 1 [very negative] to 7 [very positive]. Therefore, a positive parameter indicates less negative ratings, whereas a negative parameter corresponds to more negative ratings.

## Code availability

JavaScript codes for the encoding and memory tasks are available at https://doi.org/10.17605/OSF.IO/YJC4S.
