# Supplementary material for: The effects of shared, depression-specific, and anxiety-specific internalizing symptoms on negative and neutral episodic memories following post-learning sleep
Source: Cogn Affect Behav Neurosci. 2024 Aug 13;25(1):114–34. doi: 10.3758/s13415-024-01209-5 (PMC11805811; doi:10.3758/s13415-024-01209-5)
Supplement: Supplementary file 1 — Supplementary file1 (PDF 354 KB) [file 13415_2024_1209_MOESM1_ESM.pdf]

Descriptive Statistics

Table S1

Descriptive Statistics of Key Study Variables

		Negative scenes		Neutral scenes		Difference	
		M	*SD*	*M*	*SD*	*M*	*SD*
Encoding ratings							
Valence	Wake	2.61	0.71	4.78	0.66	2.17	0.98
	Sleep	2.77	0.70	4.76	0.61	1.98	0.81
Arousal	Wake	5.38	0.75	3.54	0.74	1.87	1.02
	Sleep	5.27	0.63	3.53	0.68	1.74	0.90
Specific memory							
Object memory	Wake	0.50	0.27	0.39	0.25	0.12	0.19
	Sleep	0.57	0.26	0.41	0.25	0.16	0.19
Background memory	Wake	0.26	0.20	0.32	0.25	-0.06	0.17
	Sleep	0.28	0.20	0.37	0.23	-0.10	0.17
Memory trade-off	Wake	0.24	0.21	0.07	0.21	0.17	0.25
	Sleep	0.29	0.22	0.04	0.20	0.25	0.26
Gist memory							
Object memory	Wake	0.40	0.24	0.36	0.22	0.04	0.18
	Sleep	0.46	0.23	0.38	0.22	0.09	0.20
Background memory	Wake	0.27	0.17	0.32	0.19	-0.05	0.11
	Sleep	0.26	0.18	0.33	0.19	-0.07	0.12
Memory trade-off	Wake	0.13	0.19	0.04	0.18	0.09	0.21
	Sleep	0.20	0.19	0.05	0.18	0.15	0.24
		**General distress**		**Anhedonia**		**Anxious arousal**	
Mini-MASQ	Wake	14.33	6.58	21.57	6.51	13.39	5.49
	Sleep	13.68	6.03	22.98	6.33	12.37	4.08

Note. Mini-MASQ, the mini version of the Mood and Anxiety Symptom Questionnaire used to measure general distress, anhedonia, and anxious arousal, calculated as the composite score of corresponding items.

For Review Only

Table S2

Bivariate Correlation Between Key Study Variables and Specific Memory

	1.	2.	3.	4.	5.	6.	7.	8.	9.	10.	11.	12.	13.	14.	15.	16.	17.	18.
1. Negative valence	1																	
2. Neutral valence	0.09	1																
3. Valence difference	-0.72***	0.63***	1															
4. Negative arousal	-0.61***	0.20**	0.62***	1														
5. Neutral arousal	0.29***	-0.30***	-0.44***	0.01	1													
6. Arousal difference	-0.64***	0.35***	0.75***	0.71***	-0.70***	1												
7. Memory for negative objects	-0.19**	0.08	0.21**	0.10	-0.32***	0.33***	1											
8. Memory for negative backgrounds	-0.15*	0.09	0.17**	0.04	-0.25***	0.21**	0.59***	1										
9. Memory for neutral objects	-0.20**	0.15*	0.26***	0.20**	-0.28***	0.36***	0.73***	0.56***	1									
10. Memory for neutral backgrounds	-0.16**	0.14*	0.22***	0.05	-0.31***	0.28***	0.68***	0.71***	0.65***	1								
11. Difference in object memory	-0.01	-0.08	-0.06	-0.11	-0.08	0.00	0.45***	0.10	-0.29***	0.10	1							
12. Difference in background memory	0.05	-0.10	-0.11	-0.02	0.13*	-0.14*	-0.25***	0.18**	-0.25***	-0.56***	-0.02	1						
13. Negative memory trade-off	-0.09	0.02	0.09	0.08	-0.16**	0.21***	0.67***	-0.20**	0.37***	0.17**	0.46***	-0.47***	1					
14. Neutral memory trade-off	-0.05	0.02	0.06	0.18**	0.01	0.12	0.10	-0.15*	0.46***	-0.38***	-0.46***	0.35***	0.25***	1				
15. Full memory trade-off	-0.04	0.00	0.03	-0.07	-0.15*	0.09	0.50***	-0.05	-0.05	0.44***	0.75***	-0.68***	0.65***	-0.57***	1			
16. General distress	-0.04	-0.01	0.03	0.04	0.07	-0.03	0.03	0.04	0.00	0.05	0.04	-0.02	0.00	-0.05	0.04	1		
17. Anhedonia	0.03	-0.25***	-0.20**	-0.06	0.15*	-0.17**	0.02	0.09	-0.02	0.03	0.06	0.06	-0.05	-0.06	0.00	0.61***	1	
18. Anxious arousal	0.13	0.01	-0.10	-0.10	0.23***	-0.25***	-0.23***	-0.19**	-0.19**	-0.16*	-0.08	0.00	-0.10	-0.03	-0.06	0.47***	0.16*	1

Note. Negative backgrounds, neutral backgrounds associated with negative objects. Neutral backgrounds, neutral backgrounds associated with neutral objects. *, p<.05. **, p<.01. ***, p<.001.

For Review Only

Table S3

Bivariate Correlation Between Key Study Variables and Gist Memory

	1.	2.	3.	4.	5.	6.	7.	8.	9.	10.	11.	12.	13.	14.	15.	16.	17.	18.
1. Negative valence	1																	
2. Neutral valence	0.09	1																
3. Valence difference	-0.72***	0.63***	1															
4. Negative arousal	-0.61***	0.20**	0.62***	1														
5. Neutral arousal	0.29***	-0.30***	-0.44***	0.01	1													
6. Arousal difference	-0.64***	0.35***	0.75***	0.71***	-0.70***	1												
7. Memory for negative objects	-0.10	0.03	0.11	0.06	-0.28***	0.27***	1											
8. Memory for negative backgrounds	-0.07	0.12	0.15*	0.07	-0.24***	0.25***	0.60***	1										
9. Memory for neutral objects	-0.11	0.13*	0.18**	0.13*	-0.24***	0.29***	0.66***	0.57***	1									
10. Memory for neutral backgrounds	-0.13*	0.16**	0.22***	0.08	-0.25***	0.28***	0.65***	0.80***	0.61***	1								
11. Difference in object memory	-0.01	-0.12	-0.08	-0.07	-0.07	0.00	0.48***	0.08	-0.34***	0.10	1							
12. Difference in background memory	0.11	-0.08	-0.14*	-0.03	0.05	-0.08	-0.17**	0.19**	-0.15*	-0.44***	-0.04	1						
13. Negative memory trade-off	-0.07	-0.07	0.00	0.01	-0.12*	0.10	0.69***	-0.17**	0.29***	0.08	0.52***	-0.38***	1					
14. Neutral memory trade-off	0.01	-0.01	-0.01	0.07	-0.02	0.06	0.12*	-0.15*	0.57***	-0.30***	-0.51***	0.28***	0.28***	1				
15. Full memory trade-off	-0.06	-0.05	0.00	-0.04	-0.09	0.04	0.49***	-0.03	-0.21***	0.31***	0.86***	-0.55***	0.63***	-0.57***	1			
16. General distress	-0.04	-0.01	0.03	0.04	0.07	-0.03	0.06	0.10	0.04	0.06	0.02	0.06	-0.02	-0.01	-0.01	1		
17. Anhedonia	0.03	-0.25***	-0.20**	-0.06	0.15*	-0.17**	0.08	0.11	-0.03	0.03	0.13*	0.11	0.00	-0.07	0.06	0.61***	1	
18. Anxious arousal	0.13	0.01	-0.10	-0.10	0.23***	-0.25***	-0.27***	-0.21**	-0.17**	-0.19**	-0.14*	0.00	-0.14*	-0.01	-0.11	0.47***	0.16*	1

Note. Negative backgrounds, neutral backgrounds associated with negative objects. Neutral backgrounds, neutral backgrounds associated with neutral objects. *, $p<.05$. **, $p<.01$. ***, $p<.001$.

For Review Only

Confirmative Factor Analyses

Table S4

Model Fit Statistics for Bifactor S-1, Bifactor, and Three-Factor Models

	**Bifactor**	**Bifactor S-1: Freed**	**Bifactor S-1: Constrained**	**Three-Factor**
RMSEA	0.06	0.08	0.08	0.09
CFI	0.91	0.86	0.85	0.79
TLI	0.89	0.83	0.83	0.77
SRMR	0.07	0.06	0.07	0.12
AIC	13582.71	13790.80	13807.30	14060.53
BIC	13952.22	14135.43	14148.38	14348.32
χ^2	$\chi^2(273)=541.93$	$\chi^2(280)=704.94$	$\chi^2(281)=720.59$	$\chi^2(296)=921.86$
Scale Correction	1.29	1.30	1.30	1.32
Model Comparison (***TRd***)		$\Delta\chi^2(7)=128.60^{***}$	$\Delta\chi^2(1)=15.65^{***}$	$\Delta\chi^2(15)=165.28^{***}$

Note. Freed, allowing depression-specific anhedonia and anxiety-specific anxious arousal factors to correlate. Constrained, constraining anhedonia and anxious arousal factors to be orthogonal. RMSEA, root-mean-square error of approximation. CFI, comparative fit index. TLI, Tucker Lewis Index. SRMR, standardized root mean-squared residual. AIC, Akaike Information Criterion. BIC, Bayesian Information Criterion. Model comparison, pairwise model comparisons with the previous (left) model. TRd, Sattora-Bentler Scaled Chi-Square Difference. ***, p<.001.

For Review Only

Table S5

Standardized CFA Results of Bifactor S-1 Modeling Freeing Specific Item-Correlation

	β with 95% CI	*S.E.*	*p*
**Common internalizing factor: general distress**			
C1. Felt tense or “high strung”	0.54 (0.40, 0.68)	0.07	<.001
C2. Felt depressed	0.77 (0.70, 0.83)	0.04	<.001
C3. Felt hopeless	0.82 (0.76, 0.88)	0.03	<.001
C4. Felt keyed up, “on edge”	0.60 (0.48, 0.72)	0.06	<.001
C5. Felt worthless	0.83 (0.76, 0.90)	0.04	<.001
C6. Felt like a failure	0.85 (0.78, 0.92)	0.04	<.001
C7. Felt uneasy	0.73 (0.65, 0.81)	0.04	<.001
C8. Felt discouraged	0.87 (0.82, 0.92)	0.03	<.001
D1. Felt withdrawn from other people	0.73 (0.65, 0.82)	0.04	<.001
D2. Felt like nothing was very enjoyable	0.78 (0.68, 0.88)	0.05	<.001
D3. Felt really happy*	0.52 (0.41, 0.62)	0.05	<.001
D4. Felt like I had a lot to look forward to*	0.38 (0.27, 0.49)	0.06	<.001
D5. Felt like I had a lot of interesting things to do*	0.36 (0.24, 0.48)	0.06	<.001
D6. Felt really lively, “up” *	0.26 (0.14, 0.38)	0.06	<.001
D7. Felt like I had a lot of energy*	0.38 (0.27, 0.50)	0.06	<.001
D8. Felt like I was having a lot of fun*	0.45 (0.34, 0.56)	0.05	<.001
A1. Was short of breath	0.33 (0.19, 0.46)	0.07	<.001
A2. Felt dizzy or lightheaded	0.32 (0.17, 0.47)	0.07	<.001
A3. Hands were cold or sweaty	0.17 (0.02, 0.32)	0.07	.026
A4. Hands were shaky	0.44 (0.28, 0.59)	0.08	<.001
A5. Had trouble swallowing	0.38 (0.20, 0.56)	0.09	<.001
A6. Had hot or cold spells	0.28 (0.12, 0.44)	0.08	.001
A7. Felt like I was choking	0.44 (0.30, 0.59)	0.07	<.001
A8. Muscles twitched or trembled	0.36 (0.20, 0.51)	0.08	<.001
A9. Was trembling or shaking	0.40 (0.23, 0.57)	0.09	<.001
A10. Had a very dry mouth	0.40 (0.23, 0.56)	0.09	<.001

For Rev

	β with 95% CI	*S.E.*	*p*
**Depression-specific factor: anhedonia**			
D1. Felt withdrawn from other people	0.02 (-0.08, 0.12)	0.05	.701
D2. Felt like nothing was very enjoyable	0.09 (-0.02, 0.20)	0.06	.124
D3. Felt really happy*	0.62 (0.53, 0.71)	0.05	<.001
D4. Felt like I had a lot to look forward to*	0.61 (0.52, 0.71)	0.05	<.001
D5. Felt like I had a lot of interesting things to do*	0.73 (0.66, 0.80)	0.04	<.001
D6. Felt really lively, "up" *	0.59 (0.48, 0.70)	0.06	<.001
D7. Felt like I had a lot of energy*	0.71 (0.64, 0.79)	0.04	<.001
D8. Felt like I was having a lot of fun*	0.72 (0.64, 0.81)	0.04	<.001
**Anxiety-specific factor: anxious arousal**			
A1. Was short of breath	0.53 (0.38, 0.67)	0.07	<.001
A2. Felt dizzy or lightheaded	0.44 (0.27, 0.62)	0.09	<.001
A3. Hands were cold or sweaty	0.42 (0.28, 0.57)	0.07	<.001
A4. Hands were shaky	0.67 (0.53, 0.80)	0.07	<.001
A5. Had trouble swallowing	0.71 (0.57, 0.84)	0.07	<.001
A6. Had hot or cold spells	0.40 (0.25, 0.55)	0.07	<.001
A7. Felt like I was choking	0.70 (0.53, 0.86)	0.09	<.001
A8. Muscles twitched or trembled	0.54 (0.41, 0.68)	0.07	<.001
A9. Was trembling or shaking	0.68 (0.53, 0.82)	0.07	<.001
A10. Had a very dry mouth	0.50 (0.35, 0.64)	0.08	<.001
**Anhedonia with anxious arousal**	-0.31 (-0.49, -0.13)	0.09	.001

Note. C, common internalizing. D, depression-specific. A, anxiety-specific. *, reverse-coded items.

Table S6

Standardized CFA Results of Bifactor S-1 Modeling Constraining Specific Item-Correlation

	β with 95% CI	*S.E.*	*p*
**Common internalizing factor: general distress**			
C1. Felt tense or “high strung”	0.54 (0.40, 0.68)	0.07	<.001
C2. Felt depressed	0.76 (0.70, 0.83)	0.04	<.001
C3. Felt hopeless	0.82 (0.76, 0.88)	0.03	<.001
C4. Felt keyed up, “on edge”	0.60 (0.47, 0.72)	0.06	<.001
C5. Felt worthless	0.83 (0.76, 0.91)	0.04	<.001
C6. Felt like a failure	0.86 (0.78, 0.93)	0.04	<.001
C7. Felt uneasy	0.73 (0.65, 0.81)	0.04	<.001
C8. Felt discouraged	0.87 (0.82, 0.92)	0.03	<.001
D1. Felt withdrawn from other people	0.73 (0.65, 0.81)	0.04	<.001
D2. Felt like nothing was very enjoyable	0.78 (0.67, 0.88)	0.05	<.001
D3. Felt really happy*	0.51 (0.40, 0.62)	0.06	<.001
D4. Felt like I had a lot to look forward to*	0.37 (0.26, 0.48)	0.06	<.001
D5. Felt like I had a lot of interesting things to do*	0.35 (0.23, 0.47)	0.06	<.001
D6. Felt really lively, “up” *	0.25 (0.13, 0.37)	0.06	<.001
D7. Felt like I had a lot of energy*	0.37 (0.26, 0.49)	0.06	<.001
D8. Felt like I was having a lot of fun*	0.44 (0.33, 0.55)	0.06	<.001
A1. Was short of breath	0.32 (0.19, 0.46)	0.07	<.001
A2. Felt dizzy or lightheaded	0.32 (0.17, 0.46)	0.07	<.001
A3. Hands were cold or sweaty	0.16 (0.02, 0.31)	0.07	.028
A4. Hands were shaky	0.43 (0.28, 0.58)	0.08	<.001
A5. Had trouble swallowing	0.38 (0.20, 0.56)	0.09	<.001
A6. Had hot or cold spells	0.27 (0.11, 0.43)	0.08	.001
A7. Felt like I was choking	0.44 (0.29, 0.58)	0.07	<.001
A8. Muscles twitched or trembled	0.35 (0.20, 0.51)	0.08	<.001
A9. Was trembling or shaking	0.39 (0.23, 0.56)	0.09	<.001
A10. Had a very dry mouth	0.39 (0.22, 0.56)	0.09	<.001

For Rev

	β with 95% CI	*S.E.*	*p*
**Depression-specific factor: anhedonia**			
D1. Felt withdrawn from other people	0.02 (-0.08, 0.12)	0.05	.701
D2. Felt like nothing was very enjoyable	0.09 (-0.02, 0.20)	0.06	.124
D3. Felt really happy*	0.62 (0.53, 0.71)	0.05	<.001
D4. Felt like I had a lot to look forward to*	0.61 (0.52, 0.71)	0.05	<.001
D5. Felt like I had a lot of interesting things to do*	0.73 (0.66, 0.80)	0.04	<.001
D6. Felt really lively, “up” *	0.59 (0.48, 0.70)	0.06	<.001
D7. Felt like I had a lot of energy*	0.71 (0.64, 0.79)	0.04	<.001
D8. Felt like I was having a lot of fun*	0.72 (0.64, 0.81)	0.04	<.001
**Anxiety-specific factor: anxious arousal**			
A1. Was short of breath	0.53 (0.38, 0.67)	0.07	<.001
A2. Felt dizzy or lightheaded	0.44 (0.27, 0.62)	0.09	<.001
A3. Hands were cold or sweaty	0.42 (0.28, 0.57)	0.07	<.001
A4. Hands were shaky	0.67 (0.53, 0.80)	0.07	<.001
A5. Had trouble swallowing	0.71 (0.57, 0.84)	0.07	<.001
A6. Had hot or cold spells	0.40 (0.25, 0.55)	0.07	<.001
A7. Felt like I was choking	0.70 (0.53, 0.86)	0.09	<.001
A8. Muscles twitched or trembled	0.54 (0.41, 0.68)	0.07	<.001
A9. Was trembling or shaking	0.68 (0.53, 0.82)	0.07	<.001
A10. Had a very dry mouth	0.50 (0.35, 0.64)	0.08	<.001

Note. C, common internalizing. D, depression-specific. A, anxiety-specific. *, reverse-coded items.

Table S7

Standardized CFA Results of Conventional Bifactor Modeling

	β with 95% CI	*S.E.*	*p*
**Common internalizing factor**			
C1. Felt tense or "high strung"	0.58 (0.43, 0.74)	0.08	<.001
C2. Felt depressed	0.76 (0.69, 0.83)	0.04	<.001
C3. Felt hopeless	0.80 (0.73, 0.87)	0.04	<.001
C4. Felt keyed up, "on edge"	0.64 (0.51, 0.77)	0.07	<.001
C5. Felt worthless	0.84 (0.76, 0.93)	0.04	<.001
C6. Felt like a failure	0.86 (0.78, 0.94)	0.04	<.001
C7. Felt uneasy	0.76 (0.67, 0.84)	0.04	<.001
C8. Felt discouraged	0.87 (0.82, 0.92)	0.02	<.001
D1. Felt withdrawn from other people	0.72 (0.63, 0.80)	0.04	<.001
D2. Felt like nothing was very enjoyable	0.76 (0.65, 0.87)	0.06	<.001
D3. Felt really happy*	0.50 (0.39, 0.60)	0.06	<.001
D4. Felt like I had a lot to look forward to*	0.36 (0.25, 0.47)	0.06	<.001
D5. Felt like I had a lot of interesting things to do*	0.33 (0.22, 0.45)	0.06	<.001
D6. Felt really lively, "up" *	0.24 (0.11, 0.36)	0.06	<.001
D7. Felt like I had a lot of energy*	0.36 (0.24, 0.47)	0.06	<.001
D8. Felt like I was having a lot of fun*	0.43 (0.32, 0.54)	0.06	<.001
A1. Was short of breath	0.32 (0.18, 0.45)	0.07	<.001
A2. Felt dizzy or lightheaded	0.30 (0.16, 0.45)	0.07	<.001
A3. Hands were cold or sweaty	0.17 (0.02, 0.32)	0.07	.024
A4. Hands were shaky	0.43 (0.29, 0.58)	0.07	<.001
A5. Had trouble swallowing	0.37 (0.20, 0.54)	0.09	<.001
A6. Had hot or cold spells	0.28 (0.13, 0.44)	0.08	<.001
A7. Felt like I was choking	0.43 (0.29, 0.57)	0.07	<.001
A8. Muscles twitched or trembled	0.35 (0.20, 0.50)	0.08	<.001
A9. Was trembling or shaking	0.40 (0.23, 0.56)	0.08	<.001
A10. Had a very dry mouth	0.40 (0.23, 0.56)	0.08	<.001
**General Distress**			

	β with 95% CI	*S.E.*	*p*
C1. Felt tense or "high strung"	0.44 (0.22, 0.66)	0.11	<.001
C2. Felt depressed	0.12 (-0.02, 0.27)	0.07	.084
C3. Felt hopeless	-0.15 (-0.29, -0.01)	0.07	.041
C4. Felt keyed up, "on edge"	0.44 (0.24, 0.65)	0.11	<.001
C5. Felt worthless	-0.42 (-0.62, -0.23)	0.10	<.001
C6. Felt like a failure	-0.31 (-0.50, -0.12)	0.10	.002
C7. Felt uneasy	0.32 (0.15, 0.49)	0.09	<.001
C8. Felt discouraged	0.05 (-0.15, 0.26)	0.10	.600
**Depression-specific factor: anhedonia**			
D1. Felt withdrawn from other people	0.06 (-0.04, 0.16)	0.05	.226
D2. Felt like nothing was very enjoyable	0.12 (0.02, 0.23)	0.05	.017
D3. Felt really happy*	0.63 (0.54, 0.72)	0.05	<.001
D4. Felt like I had a lot to look forward to*	0.63 (0.53, 0.73)	0.05	<.001
D5. Felt like I had a lot of interesting things to do*	0.75 (0.69, 0.82)	0.03	<.001
D6. Felt really lively, "up" *	0.59 (0.49, 0.70)	0.06	<.001
D7. Felt like I had a lot of energy*	0.73 (0.66, 0.80)	0.04	<.001
D8. Felt like I was having a lot of fun*	0.73 (0.65, 0.81)	0.04	<.001
**Anxiety-specific factor: anxious arousal**			
A1. Was short of breath	0.53 (0.38, 0.68)	0.08	<.001
A2. Felt dizzy or lightheaded	0.46 (0.28, 0.63)	0.09	<.001
A3. Hands were cold or sweaty	0.42 (0.27, 0.57)	0.08	<.001
A4. Hands were shaky	0.67 (0.53, 0.81)	0.07	<.001
A5. Had trouble swallowing	0.71 (0.57, 0.85)	0.07	<.001
A6. Had hot or cold spells	0.39 (0.24, 0.54)	0.07	<.001
A7. Felt like I was choking	0.70 (0.53, 0.87)	0.09	<.001
A8. Muscles twitched or trembled	0.55 (0.41, 0.68)	0.07	<.001
A9. Was trembling or shaking	0.68 (0.54, 0.82)	0.07	<.001
A10. Had a very dry mouth	0.50 (0.35, 0.65)	0.08	<.001

Note. C, common internalizing. D, depression-specific. A, anxiety-specific. *, reverse-coded items.

Table S8

Standardized CFA Results of Three-Factor Modeling

	β with 95% CI	*S.E.*	*p*
**General distress**			
C1. Felt tense or "high strung"	0.54 (0.40, 0.68)	0.07	<.001
C2. Felt depressed	0.76 (0.69, 0.83)	0.04	<.001
C3. Felt hopeless	0.82 (0.75, 0.88)	0.03	<.001
C4. Felt keyed up, "on edge"	0.59 (0.46, 0.72)	0.06	<.001
C5. Felt worthless	0.83 (0.76, 0.91)	0.04	<.001
C6. Felt like a failure	0.86 (0.78, 0.93)	0.04	<.001
C7. Felt uneasy	0.74 (0.66, 0.82)	0.04	<.001
C8. Felt discouraged	0.88 (0.83, 0.93)	0.02	<.001
**Anhedonia**			
D1. Felt withdrawn from other people	0.46 (0.33, 0.59)	0.07	<.001
D2. Felt like nothing was very enjoyable	0.54 (0.41, 0.68)	0.07	<.001
D3. Felt really happy*	0.81 (0.76, 0.87)	0.03	<.001
D4. Felt like I had a lot to look forward to*	0.71 (0.62, 0.80)	0.05	<.001
D5. Felt like I had a lot of interesting things to do*	0.79 (0.73, 0.85)	0.03	<.001
D6. Felt really lively, "up"*	0.62 (0.51, 0.72)	0.05	<.001
D7. Felt like I had a lot of energy*	0.79 (0.73, 0.85)	0.03	<.001
D8. Felt like I was having a lot of fun*	0.85 (0.80, 0.90)	0.03	<.001
**Anxious Arousal**			
A1. Was short of breath	0.62 (0.50, 0.74)	0.06	<.001
A2. Felt dizzy or lightheaded	0.55 (0.36, 0.73)	0.09	<.001
A3. Hands were cold or sweaty	0.44 (0.29, 0.60)	0.08	<.001
A4. Hands were shaky	0.80 (0.68, 0.92)	0.06	<.001
A5. Had trouble swallowing	0.80 (0.65, 0.94)	0.07	<.001
A6. Had hot or cold spells	0.49 (0.34, 0.64)	0.07	<.001
A7. Felt like I was choking	0.82 (0.69, 0.96)	0.07	<.001
A8. Muscles twitched or trembled	0.65 (0.52, 0.78)	0.06	<.001
A9. Was trembling or shaking	0.79 (0.64, 0.94)	0.08	<.001
A10. Had a very dry mouth	0.64 (0.49, 0.78)	0.08	<.001

Note. C, common internalizing. D, depression-specific. A, anxiety-specific. *, reverse-coded items.

Table S9

Power and Sensitivity Analyses of Bifactor S-1 Modeling

	M	*S.E.*	Power
**Common internalizing factor: general distress**			
C1. Felt tense or "high strung"	0.60	0.07	1.00
C2. Felt depressed	0.79	0.08	1.00
C3. Felt hopeless	0.70	0.07	1.00
C4. Felt keyed up, "on edge"	0.62	0.07	1.00
C5. Felt worthless	0.79	0.08	1.00
C6. Felt like a failure	0.82	0.08	1.00
C7. Felt uneasy	0.71	0.07	1.00
C8. Felt discouraged	0.86	0.08	1.00
D1. Felt withdrawn from other people	0.79	0.08	1.00
D2. Felt like nothing was very enjoyable	0.75	0.08	1.00
D3. Felt really happy*	0.53	0.08	1.00
D4. Felt like I had a lot to look forward to*	0.46	0.09	1.00
D5. Felt like I had a lot of interesting things to do*	0.42	0.09	1.00
D6. Felt really lively, "up" *	0.29	0.08	.93
D7. Felt like I had a lot of energy*	0.41	0.09	1.00
D8. Felt like I was having a lot of fun*	0.48	0.09	1.00
A1. Was short of breath	0.23	0.07	.89
A2. Felt dizzy or lightheaded	0.21	0.07	.83
A3. Hands were cold or sweaty	0.14	0.07	.50
A4. Hands were shaky	0.32	0.08	.99
A5. Had trouble swallowing	0.17	0.07	.68
A6. Had hot or cold spells	0.23	0.07	.88
A7. Felt like I was choking	0.23	0.07	.90
A8. Muscles twitched or trembled	0.25	0.07	.92
A9. Was trembling or shaking	0.26	0.08	.93
A10. Had a very dry mouth	0.33	0.08	.99

For Review Only

	M	*S.E.*	Power
**Depression-specific factor: anhedonia**			
D1. Felt withdrawn from other people	0.02	0.08	.07
D2. Felt like nothing was very enjoyable	0.09	0.08	.21
D3. Felt really happy*	0.63	0.08	1.00
D4. Felt like I had a lot to look forward to*	0.74	0.08	1.00
D5. Felt like I had a lot of interesting things to do*	0.83	0.08	1.00
D6. Felt really lively, “up” *	0.65	0.08	1.00
D7. Felt like I had a lot of energy*	0.76	0.08	1.00
D8. Felt like I was having a lot of fun*	0.78	0.08	1.00
**Anxiety-specific factor: anxious arousal**			
A1. Was short of breath	0.37	0.09	.98
A2. Felt dizzy or lightheaded	0.29	0.08	.94
A3. Hands were cold or sweaty	0.36	0.08	.99
A4. Hands were shaky	0.48	0.09	1.00
A5. Had trouble swallowing	0.32	0.08	.96
A6. Had hot or cold spells	0.33	0.09	.96
A7. Felt like I was choking	0.36	0.08	.98
A8. Muscles twitched or trembled	0.37	0.09	.99
A9. Was trembling or shaking	0.43	0.08	1.00
A10. Had a very dry mouth	0.40	0.09	1.00
**Correlation between latent factors**			
Anhedonia with anxious arousal	-0.31	0.10	.88
**SEM sensitivity analyses**			
General distress as a predictor	0.20	0.07	.80
Anhedonia as a predictor	0.25	0.09	.80
Anxious arousal as a predictor	0.28	0.10	.80

Note. C, common internalizing. D, depression-specific. A, anxiety-specific. *, reverse-coded items. Power, measured by the percentage of significant coefficients.

iew Only

Table S10

Measurement Invariance Across Delay conditions, Age, and Sex for Bifactor S-1 Modeling

Model	RMSEA	CFI	SC	χ^2
**Delay conditions (*n*=129 for wake and *n*=129 for sleep)**				
Configural model	0.10	0.79	1.11	$\chi^2(560)=1316.29$
Metric model	0.10	0.80	1.16	$\chi^2(601)=1311.49$
Scalar model	0.10	0.80	1.15	$\chi^2(624)=1348.36$
Configural-metric Δ	0.01	-0.01		$\chi^2(41)=32.02$
Configural-scalar Δ	0.01	-0.01		$\chi^2(64)=60.80$
Metric-scalar Δ	0.00	0.00		$\chi^2(23)=34.64$
**Age (*n*=106 for younger and *n*=152 for older)**				
Configural model	0.10	0.80	1.10	$\chi^2(560)=1259.54$
Metric model	0.09	0.82	1.15	$\chi^2(601)=1262.16$
Scalar model	0.09	0.81	1.14	$\chi^2(624)=1286.87$
Configural-metric Δ	0.01	-0.01		$\chi^2(41)=35.88$
Configural-scalar Δ	0.01	-0.01		$\chi^2(64)=56.52$
Metric-scalar Δ	0.00	0.00		$\chi^2(23)=20.91$
**Sex (*n*=134 for females and *n*=123 for males)**				
Configural model	0.09	0.82	1.14	$\chi^2(560)=1179.28$
Metric model	0.09	0.81	1.17	$\chi^2(601)=1267.69$
Scalar model	0.09	0.80	1.16	$\chi^2(624)=1324.18$
Configural-metric Δ	0.00	0.01		$\chi^2(41)=87.83^{***}$
Configural-scalar Δ	0.00	0.02		$\chi^2(64)=143.43^{***}$
Metric-scalar Δ	0.00	0.01		$\chi^2(23)=59.08^{***}$

Note. RMSEA, root-mean-square error of approximation. CFI, comparative fit index.

Younger, 18-35 years old. Older, 36-59 years old. ***, $p<.001$.

For Review Only

Structure Equation Modeling

Table S11

Standardized SEM Results of Internalizing Symptoms Measured by Bifactor S-1 Models Predicting Valence and Arousal Ratings

Outcome	Predictor	*ß with 95% CI*	*S.E.*	*p*	Adjusted q
**Valence for negative scenes**	General distress	-0.03 (-0.15, 0.10)	0.06	.682	.558
	Anhedonia	0.10 (-0.04, 0.25)	0.08	.170	.191
	Anxious arousal	0.24 (0.03, 0.44)	0.10	.023	.052
**Valence for neutral scenes**	General distress	-0.04 (-0.19, 0.10)	0.07	.562	.506
	Anhedonia	-0.35 (-0.48, -0.22)	0.07	<.001	<.001
	Anxious arousal	-0.13 (-0.29, 0.03)	0.08	.122	.157
**Arousal for negative scenes**	General distress	-0.01 (-0.16, 0.15)	0.08	.941	.706
	Anhedonia	-0.12 (-0.26, 0.03)	0.07	.114	.157
	Anxious arousal	-0.19 (-0.42, 0.04)	0.12	.103	.157
**Arousal for neutral scenes**	General distress	0.06 (-0.07, 0.20)	0.07	.346	.346
	Anhedonia	0.22 (0.08, 0.36)	0.07	.003	.009
	Anxious arousal	0.33 (0.18, 0.48)	0.08	<.001	<.001

Note. Adjusted q, two-stage controlling procedure for the false discovery rate.

Table S12

Standardized Multigroup SEM Results of Internalizing Symptoms Measured by Bifactor S-1 Models Predicting Valence and Arousal Ratings in the Wake and Sleep Conditions

Outcome	Predictor	*β with 95% CI*	*S.E.*	*p*	Adjusted q
		**Daytime Wake**			
**Valence for negative scenes**	General distress	-0.04 (-0.23, 0.15)	0.10	.662	.542
	Anhedonia	0.07 (-0.16, 0.30)	0.12	.564	.542
	Anxious arousal	0.27 (0.04, 0.51)	0.12	.025	.056
**Valence for neutral scenes**	General distress	-0.21 (-0.38, -0.04)	0.09	.016	.041
	Anhedonia	-0.41 (-0.57, -0.25)	0.08	<.001	<.001
	Anxious arousal	-0.24 (-0.37, -0.11)	0.07	<.001	<.001
**Arousal for negative scenes**	General distress	-0.06 (-0.27, 0.16)	0.11	.600	.542
	Anhedonia	-0.06 (-0.28, 0.17)	0.11	.631	.542
	Anxious arousal	-0.26 (-0.54, 0.02)	0.14	.071	.128
**Arousal for neutral scenes**	General distress	0.10 (-0.09, 0.29)	0.10	.299	.359
	Anhedonia	0.08 (-0.11, 0.27)	0.10	.421	.446
	Anxious arousal	0.27 (0.10, 0.45)	0.09	.002	.009
		**Nighttime Sleep**			
**Valence for negative scenes**	General distress	-0.01 (-0.18, 0.16)	0.09	.898	.674
	Anhedonia	0.12 (-0.07, 0.31)	0.10	.215	.298
	Anxious arousal	0.19 (-0.14, 0.52)	0.17	.251	.323
**Valence for neutral scenes**	General distress	0.20 (0.01, 0.38)	0.09	.035	.070
	Anhedonia	-0.28 (-0.47, -0.09)	0.10	.004	.014
	Anxious arousal	0.13 (-0.04, 0.30)	0.09	.139	.209
**Arousal for negative scenes**	General distress	0.08 (-0.12, 0.28)	0.10	.418	.446
	Anhedonia	-0.17 (-0.36, 0.03)	0.10	.090	.147
	Anxious arousal	-0.05 (-0.28, 0.19)	0.12	.693	.542
**Arousal for neutral scenes**	General distress	0.04 (-0.12, 0.19)	0.08	.639	.542
	Anhedonia	0.37 (0.20, 0.54)	0.09	<.001	<.001
	Anxious arousal	0.35 (0.10, 0.61)	0.13	.007	.021

Note. Adjusted q, two-stage controlling procedure for the false discovery rate.

Table S13

Standardized SEM Results of Internalizing Symptoms Measured by Bifactor S-1 Models Predicting the Difference in Valence and Arousal Ratings Between Negative Scenes and Neutral Scenes

Outcome	Predictor	*β with 95% CI*	*S.E.*	*p*	Adjusted q
**Difference in valence**	General distress	-0.01 (-0.16, 0.14)	0.08	.875	.292
	Anhedonia	-0.33 (-0.46, -0.20)	0.07	<.001	<.001
	Anxious arousal	-0.28 (-0.47, -0.09)	0.10	.004	.002
**Difference in arousal**	General distress	-0.06 (-0.20, 0.09)	0.07	.456	.182
	Anhedonia	-0.25 (-0.40, -0.10)	0.08	.001	.001
	Anxious arousal	-0.39 (-0.58, -0.20)	0.10	<.001	<.001

Note. Adjusted q, two-stage controlling procedure for the false discovery rate.

eview Only

Table S14

Standardized Multigroup SEM Results of Internalizing Symptoms Measured by Bifactor S-1 Models Predicting Difference in Valence and Arousal Ratings in the Wake and Sleep Conditions

Outcome	Predictor	*β with 95% CI*	*S.E.*	*p*	Adjusted q
		**Daytime Wake**			
**Difference in valence**	General distress	-0.11 (-0.30, 0.08)	0.10	.247	.212
	Anhedonia	-0.33 (-0.52, -0.14)	0.10	.001	.001
	Anxious arousal	-0.37 (-0.56, -0.18)	0.10	<.001	<.001
**Difference in arousal**	General distress	-0.12 (-0.32, 0.09)	0.10	.273	.212
	Anhedonia	-0.10 (-0.32, 0.12)	0.11	.385	.270
	Anxious arousal	-0.41 (-0.60, -0.22)	0.10	<.001	<.001
		**Nighttime Sleep**			
**Difference in valence**	General distress	0.16 (-0.02, 0.33)	0.09	.082	.082
	Anhedonia	-0.32 (-0.50, -0.14)	0.09	.001	.001
	Anxious arousal	-0.07 (-0.34, 0.21)	0.14	.632	.402
**Difference in arousal**	General distress	0.02 (-0.16, 0.20)	0.09	.800	.467
	Anhedonia	-0.39 (-0.58, -0.20)	0.10	<.001	<.001
	Anxious arousal	-0.30 (-0.59, -0.01)	0.15	.041	.048

Note. Adjusted q, two-stage controlling procedure for the false discovery rate.

Table S15

Standardized SEM Results of Internalizing Symptoms Measured by Bifactor S-1 Models Predicting Specific Recognition Memory

Outcome for Memory	Predictor	*β with 95% CI*	*S.E.*	*p*	Adjusted q
**Negative objects**	General distress	0.01 (-0.11, 0.13)	0.06	.858	.624
	Anhedonia	-0.06 (-0.20, 0.09)	0.07	.465	.413
	Anxious arousal	-0.30 (-0.46, -0.15)	0.08	<.001	<.001
**Negative backgrounds**	General distress	0.06 (-0.07, 0.20)	0.07	.334	.413
	Anhedonia	0.00 (-0.14, 0.14)	0.07	.998	.665
	Anxious arousal	-0.26 (-0.39, -0.13)	0.07	<.001	<.001
**Neutral objects**	General distress	-0.01 (-0.13, 0.10)	0.06	.846	.624
	Anhedonia	-0.06 (-0.20, 0.09)	0.07	.430	.413
	Anxious arousal	-0.24 (-0.38, -0.11)	0.07	.001	.002
**Neutral backgrounds**	General distress	0.05 (-0.07, 0.17)	0.06	.439	.413
	Anhedonia	-0.06 (-0.19, 0.08)	0.07	.423	.413
	Anxious arousal	-0.24 (-0.38, -0.10)	0.07	.001	.002

Note. Adjusted q, two-stage controlling procedure for the false discovery rate.

ly

Table S16

Standardized Multigroup SEM Results of Internalizing Symptoms Measured by Bifactor S-1 Models Predicting Specific Recognition memory in the Wake and Sleep Conditions

Outcome	Predictor	*β with 95% CI*	*S.E.*	*p*	Adjusted q
		**Daytime Wake**			
**Negative objects**	General distress	-0.01 (-0.18, 0.16)	0.09	.886	.738
	Anhedonia	-0.04 (-0.24, 0.17)	0.10	.708	.708
	Anxious arousal	-0.34 (-0.51, -0.16)	0.09	<.001	<.001
**Negative backgrounds**	General distress	0.02 (-0.15, 0.19)	0.09	.801	.728
	Anhedonia	-0.06 (-0.25, 0.12)	0.10	.492	.562
	Anxious arousal	-0.30 (-0.47, -0.13)	0.09	<.001	<.001
**Neutral objects**	General distress	-0.11 (-0.27, 0.05)	0.08	.170	.298
	Anhedonia	0.03 (-0.18, 0.23)	0.10	.800	.728
	Anxious arousal	-0.26 (-0.41, -0.11)	0.08	.001	.005
**Neutral backgrounds**	General distress	0.02 (-0.16, 0.20)	0.09	.839	.730
	Anhedonia	-0.19 (-0.38, -0.01)	0.10	.040	.143
	Anxious arousal	-0.30 (-0.45, -0.14)	0.08	<.001	<.001
		**Nighttime Sleep**			
**Negative objects**	General distress	0.06 (-0.10, 0.23)	0.08	.450	.562
	Anhedonia	-0.09 (-0.29, 0.11)	0.10	.388	.517
	Anxious arousal	-0.22 (-0.47, 0.02)	0.12	.073	.209
**Negative backgrounds**	General distress	0.12 (-0.06, 0.32)	0.10	.196	.302
	Anhedonia	0.07 (-0.13, 0.27)	0.10	.506	.562
	Anxious arousal	-0.20 (-0.39, -0.01)	0.10	.043	.143
**Neutral objects**	General distress	0.12 (-0.04, 0.27)	0.08	.141	.298
	Anhedonia	-0.11 (-0.30, 0.08)	0.10	.242	.346
	Anxious arousal	-0.15 (-0.35, 0.04)	0.10	.129	.298
**Neutral backgrounds**	General distress	0.11 (-0.04, 0.27)	0.08	.149	.298
	Anhedonia	0.05 (-0.16, 0.25)	0.10	.659	.694
	Anxious arousal	-0.16 (-0.40, 0.07)	0.12	.179	.298

Note. Adjusted q, two-stage controlling procedure for the false discovery rate.

Table S17

Standardized SEM Results of Internalizing Symptoms Measured by Bifactor S-1 Models Predicting Difference in Specific Recognition Memory Between Negative and Neutral Scene Components

Outcome	Predictor	*β with 95% CI*	*S.E.*	*p*	Adjusted q
**Difference in object memory**	General distress	0.03 (-0.08, 0.14)	0.06	.575	.862
	Anhedonia	0.00 (-0.14, 0.13)	0.07	.966	.966
	Anxious arousal	-0.11 (-0.24, 0.01)	0.06	.084	.504
**Difference in background memory**	General distress	0.01 (-0.12, 0.14)	0.06	.882	.966
	Anhedonia	0.08 (-0.05, 0.21)	0.07	.233	.699
	Anxious arousal	0.04 (-0.09, 0.16)	0.06	.553	.862

Note. Adjusted q, two-stage controlling procedure for the false discovery rate.

For Review Only

Table S18

Standardized Multigroup SEM Results of Internalizing Symptoms Measured by Bifactor S-1 Models Predicting Difference in Specific Recognition Memory Between Negative and Neutral Scene Components in the Wake and Sleep Conditions

Outcome	Predictor	*β with 95% CI*	*S.E.*	*p*	Adjusted q
		**Daytime Wake**			
**Difference in object memory**	General distress	0.13 (-0.04, 0.29)	0.08	.124	.496
	Anhedonia	-0.09 (-0.28, 0.09)	0.09	.333	.666
	Anxious arousal	-0.14 (-0.30, 0.02)	0.08	.081	.486
**Difference in background memory**	General distress	0.00 (-0.17, 0.18)	0.09	.954	.969
	Anhedonia	0.19 (0.00, 0.39)	0.10	.051	.486
	Anxious arousal	0.08 (-0.06, 0.23)	0.07	.277	.666
		**Nighttime Sleep**			
**Difference in object memory**	General distress	-0.06 (-0.21, 0.09)	0.08	.419	.718
	Anhedonia	0.02 (-0.17, 0.21)	0.10	.834	.969
	Anxious arousal	-0.12 (-0.34, 0.10)	0.11	.296	.666
**Difference in background memory**	General distress	0.00 (-0.19, 0.18)	0.10	.969	.969
	Anhedonia	0.02 (-0.16, 0.20)	0.09	.827	.969
	Anxious arousal	-0.02 (-0.22, 0.19)	0.11	.875	.969

Note. Adjusted q, two-stage controlling procedure for the false discovery rate.

Table S19

Standardized SEM Results of Internalizing Symptoms Measured by Bifactor S-1 Models Predicting Specific Negative and Neutral Memory Trade-Off Effects

Outcome	Predictor	*β with 95% CI*	*S.E.*	*p*	Adjusted q
**Negative memory trade-off**	General distress	0.04 (-0.08, 0.17)	0.07	.493	.740
	Anhedonia	-0.07 (-0.21, 0.07)	0.07	.344	.688
	Anxious arousal	-0.13 (-0.28, 0.01)	0.07	.068	.408
**Neutral memory trade-off**	General distress	0.07 (-0.05, 0.19)	0.06	.245	.688
	Anhedonia	0.00 (-0.17, 0.16)	0.08	.951	.951
	Anxious arousal	-0.01 (-0.14, 0.12)	0.06	.881	.951

Note. Adjusted q, two-stage controlling procedure for the false discovery rate.

review Only

Table S20

Standardized Multigroup SEM Results of Internalizing Symptoms Measured by Bifactor S-1 Models Predicting Specific Negative and Neutral Memory Trade-Off Effects in the Wake and Sleep Conditions

Outcome	Predictor	*β with 95% CI*	*S.E.*	*p*	Adjusted q
		**Daytime Wake**			
**Negative memory trade-off**	General distress	0.04 (-0.12, 0.20)	0.08	.632	.940
	Anhedonia	0.01 (-0.20, 0.22)	0.11	.926	.940
	Anxious arousal	-0.14 (-0.32, 0.04)	0.09	.116	.278
**Neutral memory trade-off**	General distress	0.16 (0.00, 0.31)	0.08	.048	.236
	Anhedonia	0.26 (0.03, 0.48)	0.12	.025	.236
	Anxious arousal	0.05 (-0.08, 0.19)	0.07	.433	.742
		**Nighttime Sleep**			
**Negative memory trade-off**	General distress	0.04 (-0.16, 0.24)	0.10	.720	.940
	Anhedonia	-0.16 (-0.35, 0.02)	0.09	.079	.237
	Anxious arousal	-0.09 (-0.31, 0.13)	0.11	.419	.742
**Neutral memory trade-off**	General distress	-0.01 (-0.18, 0.16)	0.09	.886	.940
	Anhedonia	-0.19 (-0.39, 0.01)	0.10	.059	.236
	Anxious arousal	-0.01 (-0.23, 0.22)	0.12	.940	.940

Note. Adjusted q, two-stage controlling procedure for the false discovery rate.

Table S21

Standardized SEM Results of Internalizing Symptoms Measured by Bifactor S-1 Models Predicting Specific Full Memory Trade-Off Effects

Outcome	Predictor	*β with 95% CI*	*S.E.*	*p*	Adjusted q
**Full memory trade-off**	General distress	0.02 (-0.10, 0.13)	0.06	.790	.790
	Anhedonia	-0.06 (-0.19, 0.08)	0.07	.427	.640
	Anxious arousal	-0.11 (-0.25, 0.04)	0.07	.140	.420

Note. Adjusted q, two-stage controlling procedure for the false discovery rate.

For Review Only

Table S22

Standardized Multigroup SEM Results of Internalizing Symptoms Measured by Bifactor S-1 Models Predicting Specific Full Trade-Off Effects in the Wake and Sleep Conditions

Outcome	Predictor	*β with 95% CI*	*S.E.*	*p*	Adjusted q
		**Daytime Wake**			
**Full memory trade-off**	General distress	0.09 (-0.07, 0.26)	0.08	.271	.542
	Anhedonia	-0.20 (-0.38, -0.01)	0.10	.038	.204
	Anxious arousal	-0.16 (-0.34, 0.01)	0.09	.068	.204
		**Nighttime Sleep**			
**Full memory trade-off**	General distress	-0.04 (-0.20, 0.12)	0.08	.614	.737
	Anhedonia	0.00 (-0.19, 0.19)	0.10	.985	.985
	Anxious arousal	-0.07 (-0.32, 0.18)	0.13	.570	.737

Note. Adjusted q, two-stage controlling procedure for the false discovery rate.

iew Only

Table S23

Standardized SEM Results of Internalizing Symptoms Measured by Bifactor S-1 Models Predicting Gist Recognition Memory

Outcome for Memory	Predictor	*β with 95% CI*	*S.E.*	*p*	Adjusted q
**Negative objects**	General distress	0.03 (-0.10, 0.16)	0.07	.631	.561
	Anhedonia	0.01 (-0.13, 0.15)	0.07	.898	.599
	Anxious arousal	-0.36 (-0.49, -0.23)	0.06	<.001	<.001
**Negative backgrounds**	General distress	0.10 (-0.03, 0.22)	0.06	.125	.200
	Anhedonia	-0.02 (-0.16, 0.12)	0.07	.777	.565
	Anxious arousal	-0.33 (-0.47, -0.20)	0.07	<.001	<.001
**Neutral objects**	General distress	0.02 (-0.10, 0.14)	0.06	.702	.562
	Anhedonia	-0.09 (-0.23, 0.05)	0.07	.226	.301
	Anxious arousal	-0.24 (-0.39, -0.09)	0.07	.002	.004
**Neutral backgrounds**	General distress	0.04 (-0.07, 0.16)	0.06	.445	.445
	Anhedonia	-0.06 (-0.20, 0.08)	0.07	.413	.445
	Anxious arousal	-0.27 (-0.40, -0.14)	0.07	<.001	<.001

Note. Adjusted q, two-stage controlling procedure for the false discovery rate.

nly

Table S24

Standardized Multigroup SEM Results of Internalizing Symptoms Measured by Bifactor S-1 Models Predicting Gist Recognition memory in the Wake and Sleep Conditions

Outcome for memory	Predictor	*β with 95% CI*	*S.E.*	*p*	Adjusted q
		**Daytime Wake**			
**Negative objects**	General distress	0.05 (-0.12, 0.22)	0.09	.574	.542
	Anhedonia	0.07 (-0.14, 0.27)	0.10	.507	.507
	Anxious arousal	-0.35 (-0.51, -0.20)	0.08	<.001	<.001
**Negative backgrounds**	General distress	0.06 (-0.10, 0.22)	0.08	.467	.496
	Anhedonia	0.04 (-0.15, 0.23)	0.10	.681	.551
	Anxious arousal	-0.34 (-0.50, -0.17)	0.09	<.001	<.001
**Neutral objects**	General distress	-0.08 (-0.23, 0.07)	0.08	.305	.399
	Anhedonia	0.00 (-0.19, 0.19)	0.10	.989	.701
	Anxious arousal	-0.22 (-0.37, -0.07)	0.08	.004	.010
**Neutral backgrounds**	General distress	0.00 (-0.17, 0.16)	0.08	.972	.701
	Anhedonia	0.00 (-0.18, 0.18)	0.09	.987	.701
	Anxious arousal	-0.24 (-0.41, -0.08)	0.08	.003	.009
		**Nighttime Sleep**			
**Negative objects**	General distress	0.04 (-0.14, 0.22)	0.09	.649	.551
	Anhedonia	-0.09 (-0.29, 0.11)	0.10	.389	.441
	Anxious arousal	-0.36 (-0.55, -0.17)	0.10	<.001	<.001
**Negative backgrounds**	General distress	0.14 (-0.02, 0.31)	0.08	.081	.162
	Anhedonia	-0.05 (-0.24, 0.15)	0.10	.641	.551
	Anxious arousal	-0.31 (-0.47, -0.15)	0.08	<.001	<.001
**Neutral objects**	General distress	0.16 (-0.03, 0.36)	0.10	.096	.163
	Anhedonia	-0.17 (-0.37, 0.02)	0.10	.086	.162
	Anxious arousal	-0.21 (-0.49, 0.07)	0.14	.149	.230
**Neutral backgrounds**	General distress	0.10 (-0.04, 0.24)	0.07	.179	.254
	Anhedonia	-0.10 (-0.31, 0.10)	0.10	.337	.409
	Anxious arousal	-0.27 (-0.46, -0.09)	0.09	.003	.009

Note. Adjusted q, two-stage controlling procedure for the false discovery rate.

Table S25

Standardized SEM Results of Internalizing Symptoms Measured by Bifactor S-1 Models Predicting Difference in Gist Recognition Memory Between Negative and Neutral Scene Components

Outcome	Predictor	*β with 95% CI*	*S.E.*	*p*	Adjusted q
**Difference in object memory**	General distress	0.01 (-0.13, 0.16)	0.07	.873	.873
	Anhedonia	0.11 (-0.03, 0.26)	0.07	.118	.354
	Anxious arousal	-0.17 (-0.33, -0.01)	0.08	.042	.252
**Difference in background memory**	General distress	0.07 (-0.04, 0.18)	0.06	.194	.388
	Anhedonia	0.07 (-0.07, 0.21)	0.07	.343	.515
	Anxious arousal	-0.04 (-0.17, 0.09)	0.07	.529	.635

Note. Adjusted q, two-stage controlling procedure for the false discovery rate.

For Review Only

Table S26

Standardized Multigroup SEM Results of Internalizing Symptoms Measured by Bifactor S-1 Models Predicting Difference in Gist Recognition Memory Between Negative and Neutral Scene Components in the Wake and Sleep Conditions

Outcome	Predictor	*β with 95% CI*	*S.E.*	*p*	Adjusted q
		**Daytime Wake**			
**Difference in object memory**	General distress	0.16 (0.00, 0.32)	0.08	.052	.432
	Anhedonia	0.09 (-0.12, 0.29)	0.11	.425	.567
	Anxious arousal	-0.18 (-0.39, 0.04)	0.11	.108	.432
**Difference in background memory**	General distress	0.10 (-0.05, 0.25)	0.08	.177	.466
	Anhedonia	0.06 (-0.13, 0.25)	0.10	.543	.605
	Anxious arousal	-0.10 (-0.25, 0.05)	0.08	.194	.466
		**Nighttime Sleep**			
**Difference in object memory**	General distress	-0.13 (-0.35, 0.09)	0.11	.240	.480
	Anhedonia	0.08 (-0.10, 0.27)	0.10	.383	.567
	Anxious arousal	-0.21 (-0.44, 0.02)	0.12	.075	.432
**Difference in background memory**	General distress	0.05 (-0.11, 0.20)	0.08	.555	.605
	Anhedonia	0.10 (-0.10, 0.30)	0.10	.327	.561
	Anxious arousal	0.01 (-0.18, 0.20)	0.10	.905	.905

Note. Adjusted q, two-stage controlling procedure for the false discovery rate.

Table S27

Standardized SEM Results of Internalizing Symptoms Measured by Bifactor S-1 Models Predicting Gist Negative and Neutral Memory Trade-Off Effects

Outcome	Predictor	*β with 95% CI*	*S.E.*	*p*	Adjusted q
**Negative memory trade-off**	General distress	0.05 (-0.08, 0.19)	0.07	.452	.907
	Anhedonia	0.03 (-0.11, 0.16)	0.07	.700	.907
	Anxious arousal	-0.13 (-0.27, 0.01)	0.07	.060	.360
**Neutral memory trade-off**	General distress	0.02 (-0.11, 0.15)	0.06	.756	.907
	Anhedonia	-0.04 (-0.19, 0.10)	0.07	.529	.907
	Anxious arousal	0.00 (-0.14, 0.15)	0.07	.964	.964

Note. Adjusted q, two-stage controlling procedure for the false discovery rate.

review Only

Table S28

Standardized Multigroup SEM Results of Internalizing Symptoms Measured by Bifactor S-1 Models Predicting Gist Negative and Neutral Memory Trade-Off Effects in the Wake and Sleep Conditions

Outcome	Predictor	*β with 95% CI*	*S.E.*	*p*	Adjusted q
		**Daytime Wake**			
**Negative memory trade-off**	General distress	0.01 (-0.16, 0.17)	0.09	.938	.994
	Anhedonia	0.04 (-0.16, 0.24)	0.10	.676	.911
	Anxious arousal	-0.11 (-0.30, 0.08)	0.10	.255	.818
**Neutral memory trade-off**	General distress	0.10 (-0.04, 0.24)	0.07	.166	.818
	Anhedonia	0.00 (-0.21, 0.21)	0.11	.994	.994
	Anxious arousal	0.00 (-0.17, 0.17)	0.09	.969	.994
		**Nighttime Sleep**			
**Negative memory trade-off**	General distress	0.08 (-0.14, 0.29)	0.11	.477	.818
	Anhedonia	-0.07 (-0.25, 0.11)	0.09	.454	.818
	Anxious arousal	-0.17 (-0.37, 0.03)	0.10	.090	.818
**Neutral memory trade-off**	General distress	-0.09 (-0.30, 0.12)	0.11	.408	.818
	Anhedonia	-0.10 (-0.30, 0.10)	0.10	.338	.818
	Anxious arousal	0.06 (-0.21, 0.32)	0.14	.683	.911

Note. Adjusted q, two-stage controlling procedure for the false discovery rate.

Table S29

Standardized SEM Results of Internalizing Symptoms Measured by Bifactor S-1 Models Predicting Gist Full Memory Trade-Off Effects

Outcome	Predictor	*β with 95% CI*	*S.E.*	*p*	Adjusted q
**Full memory trade-off**	General distress	0.03 (-0.11, 0.16)	0.07	.686	.686
	Anhedonia	0.06 (-0.07, 0.19)	0.07	.383	.575
	Anxious arousal	-0.12 (-0.28, 0.04)	0.08	.149	.447

Note. Adjusted q, two-stage controlling procedure for the false discovery rate.

For Review Only

Table S30

Standardized Multigroup SEM Results of Internalizing Symptoms Measured by Bifactor S-1 Models Predicting Gist Full Trade-Off Effects in the Wake and Sleep Conditions

Outcome	Predictor	*β with 95% CI*	*S.E.*	*p*	Adjusted q
		**Daytime Wake**			
**Full memory trade-off**	General distress	0.08 (-0.08, 0.24)	0.08	.320	.610
	Anhedonia	0.04 (-0.16, 0.24)	0.10	.698	.838
	Anxious arousal	-0.10 (-0.33, 0.13)	0.12	.407	.610
		**Nighttime Sleep**			
**Full memory trade-off**	General distress	-0.13 (-0.33, 0.07)	0.10	.195	.585
	Anhedonia	0.02 (-0.16, 0.20)	0.09	.841	.841
	Anxious arousal	-0.18 (-0.37, 0.02)	0.10	.076	.456

Note. Adjusted q, two-stage controlling procedure for the false discovery rate.

iew Only

Wald Tests of Effect Sizes

Table S31

Wald's Comparisons of Effect Sizes for Valence and Arousal Ratings between Dimensions of Internalizing Symptoms, and between Daytime Wake and Nighttime Sleep Conditions

Outcome	$\Delta\beta$	χ^2	*p*	$\Delta\beta$	χ^2	*p*
	**General distress vs. anhedonia**			**General distress: wake vs. sleep**		
Valence for negative scenes	-0.13	2.00	.182	-0.03	0.00	.984
Valence for neutral scenes	0.30	9.95	.002	-0.40	8.46	.004
Difference in valence	0.32	9.71	.002	-0.27	0.01	.917
Arousal for negative scenes	0.11	1.01	.315	-0.14	0.84	.359
Arousal for neutral scenes	-0.16	3.04	.081	0.06	1.51	.219
Difference in arousal	0.20	3.75	.053	-0.14	0.54	.463
	**General distress vs. anxious arousal**			**Anhedonia: wake vs. sleep**		
Valence for negative scenes	-0.26	5.01	.025	-0.05	0.04	.838
Valence for neutral scenes	0.08	0.69	.406	-0.13	1.55	.213
Difference in valence	0.27	6.75	.009	-0.01	0.45	.504
Arousal for negative scenes	0.18	2.30	.129	0.11	0.14	.705
Arousal for neutral scenes	-0.27	6.87	.009	-0.29	4.26	.039
Difference in arousal	0.33	11.34	.001	0.29	2.71	.100
	**Anhedonia vs. anxious arousal**			**Anxious arousal: wake vs. sleep**		
Valence for negative scenes	-0.13	1.35	.246	0.08	0.48	.489
Valence for neutral scenes	-0.22	6.05	.014	-0.37	12.84	.000
Difference in valence	-0.05	0.27	.601	-0.30	5.46	.019
Arousal for negative scenes	0.07	0.42	.518	-0.21	2.12	.146
Arousal for neutral scenes	-0.11	1.59	.207	-0.08	0.01	.936
Difference in arousal	0.14	1.83	.176	-0.11	1.64	.201

Note. $\Delta\beta$, difference in standardized beta coefficients between general distress, anhedonia, and anxious arousal, and between daytime wake and nighttime sleep conditions.

For Review Only

Table S32

Wald's Comparisons of Effect Sizes for Specific Recognition Memory between Dimensions of Internalizing Symptoms, and between Daytime Wake and Nighttime Sleep Conditions

Outcome	$\Delta\beta$	χ^2	p	$\Delta\beta$	χ^2	p
	**General distress vs. anhedonia**			**General distress: wake vs. sleep**		
Memory for negative objects	0.07	0.43	.514	-0.08	1.00	.318
Memory for neutral objects	0.05	0.23	.630	-0.23	4.95	.026
Difference in object memory	0.03	0.14	.711	0.19	0.36	.550
Memory for negative backgrounds	0.07	0.43	.512	-0.10	1.14	.285
Memory for neutral backgrounds	0.11	1.18	.278	-0.09	0.88	.349
Difference in background memory	-0.07	0.52	.472	0.01	0.01	.914
	**General distress vs. anxious arousal**			**Anhedonia: wake vs. sleep**		
Memory for negative objects	0.31	10.34	.001	0.05	0.05	.819
Memory for neutral objects	0.23	6.91	.009	0.14	0.81	.367
Difference in object memory	0.14	2.42	.120	-0.11	0.69	.408
Memory for negative backgrounds	0.33	11.34	.001	-0.13	1.09	.296
Memory for neutral backgrounds	0.29	9.20	.002	-0.24	3.30	.070
Difference in background memory	-0.03	0.07	.786	0.17	1.80	.179
	**Anhedonia vs. anxious arousal**			**Anxious arousal: wake vs. sleep**		
Memory for negative objects	0.25	9.95	.002	-0.11	1.35	.245
Memory for neutral objects	0.19	6.23	.013	-0.11	1.43	.231
Difference in object memory	0.11	2.32	.128	-0.03	0.13	.719
Memory for negative backgrounds	0.26	12.75	.000	-0.10	1.30	.255
Memory for neutral backgrounds	0.19	6.25	.012	-0.13	2.07	.151
Difference in background memory	0.04	0.41	.522	0.10	0.78	.377

Note. $\Delta\beta$, difference in standardized beta coefficients between general distress, anhedonia, and anxious arousal, and between daytime wake and nighttime sleep conditions.

For Review Only

Table S33

Wald's Comparisons of Effect Sizes for Specific Memory Trade-Off between Dimensions of Internalizing Symptoms, and between Daytime Wake and Nighttime Sleep Conditions

Outcome	$\Delta\beta$	χ^2	p	$\Delta\beta$	χ^2	p
	**General distress vs. anhedonia**			**General distress: sleep vs. wake**		
Negative memory trade-off	0.11	1.44	.230	0.00	0.38	.540
Neutral memory trade-off	0.08	0.56	.453	0.17	1.36	.243
Full memory trade-off	0.07	0.52	.470	0.13	0.83	.362
	**General distress vs. anxious arousal**			**Anhedonia: sleep vs. wake**		
Negative memory trade-off	0.18	3.87	.049	0.18	1.50	.220
Neutral memory trade-off	0.08	1.02	.314	0.45	7.79	.005
Full memory trade-off	0.12	1.48	.225	-0.20	2.08	.150
	**Anhedonia vs. anxious arousal**			**Anxious arousal: sleep vs. wake**		
Negative memory trade-off	0.07	0.73	.394	-0.05	0.24	.622
Neutral memory trade-off	0.01	0.00	.953	0.06	0.30	.587
Full memory trade-off	0.05	0.44	.505	-0.09	0.55	.457

Note. $\Delta\beta$, difference in standardized beta coefficients between general distress, anhedonia, and anxious arousal, and between daytime wake and nighttime sleep conditions.

For Review Only

Table S34

Wald's Comparisons of Effect Sizes for Gist Recognition Memory between Dimensions of Internalizing Symptoms, and between Daytime Wake and Nighttime Sleep Conditions

Outcome	$\Delta\beta$	χ^2	*p*	$\Delta\beta$	χ^2	*p*
	**General distress vs. anhedonia**			**General distress: wake vs. sleep**		
Memory for negative objects	0.02	0.05	.816	0.01	0.22	.643
Memory for neutral objects	0.11	1.40	.237	-0.25	5.36	.021
Difference in object memory	-0.10	0.93	.336	0.29	0.05	.828
Memory for negative backgrounds	0.12	1.51	.219	-0.08	1.27	.261
Memory for neutral backgrounds	0.10	1.18	.277	-0.10	1.36	.243
Difference in background memory	0.00	0.00	.968	0.06	1.30	.254
	**General distress vs. anxious arousal**			**Anhedonia: wake vs. sleep**		
Memory for negative objects	0.39	17.34	.000	0.16	0.90	.343
Memory for neutral objects	0.26	8.28	.004	0.17	1.27	.259
Difference in object memory	0.18	3.29	.070	0.00	0.01	.922
Memory for negative backgrounds	0.43	24.29	.000	0.09	0.25	.614
Memory for neutral backgrounds	0.32	13.04	.000	0.10	0.33	.566
Difference in background memory	0.11	1.40	.237	-0.04	0.08	.784
	**Anhedonia vs. anxious arousal**			**Anxious arousal: wake vs. sleep**		
Memory for negative objects	0.37	23.10	.000	0.01	0.29	.589
Memory for neutral objects	0.15	3.43	.064	-0.01	0.18	.672
Difference in object memory	0.28	9.15	.003	0.03	0.00	.978
Memory for negative backgrounds	0.31	16.38	.000	-0.03	0.54	.462
Memory for neutral backgrounds	0.21	8.22	.004	0.03	0.08	.773
Difference in background memory	0.11	2.16	.142	-0.11	0.65	.420

Note. $\Delta\beta$, difference in standardized beta coefficients between general distress, anhedonia, and anxious arousal, and between daytime wake and nighttime sleep conditions.

For Review Only

Table S35

Wald's Comparisons of Effect Sizes for Gist Memory Trade-Off between Dimensions of Internalizing Symptoms, and between Daytime Wake and Nighttime Sleep Conditions

Outcome	$\Delta\beta$	χ^2	*p*	$\Delta\beta$	χ^2	*p*
	**General distress vs. anhedonia**			**General distress: sleep vs. wake**		
Negative memory trade-off	0.03	0.07	.789	-0.07	0.28	.600
Neutral memory trade-off	0.07	0.41	.524	0.19	0.04	.848
Full memory trade-off	-0.03	0.11	.738	0.21	0.09	.760
	**General distress vs. anxious arousal**			**Anhedonia: sleep vs. wake**		
Negative memory trade-off	0.18	4.80	.028	0.11	0.59	.443
Neutral memory trade-off	0.02	0.02	.880	0.10	0.49	.482
Full memory trade-off	0.15	1.62	.203	0.02	0.00	.976
	**Anhedonia vs. anxious arousal**			**Anxious arousal: sleep vs. wake**		
Negative memory trade-off	0.16	3.61	.057	0.06	0.03	.863
Neutral memory trade-off	-0.05	0.33	.566	-0.06	0.09	.770
Full memory trade-off	0.18	3.67	.056	0.08	0.14	.709

Note. $\Delta\beta$, difference in standardized beta coefficients between general distress, anhedonia, and anxious arousal, and between daytime wake and nighttime sleep conditions.

For Review Only

Structure Equation Modeling with Covariates

Table S36

Standardized SEM Results of Internalizing Symptoms Measured by Bifactor S-1 Models Predicting the Difference in Valence and Arousal Ratings Between Negative Scenes and Neutral Scenes Controlling for Age, Sex, and Psychomotor Vigilance Alertness

Outcome	Predictor	*β with 95% CI*	*S.E.*	*p*	Adjusted q
**Difference in valence**	General distress	-0.02 (-0.17, 0.13)	0.07	.778	.479
	Anhedonia	0.35 (0.23, 0.48)	0.06	<.001	<.001
	Anxious arousal	0.19 (0.00, 0.39)	0.10	.057	.051
	Age	-0.12 (-0.22, -0.01)	0.05	.026	.030
	Sex	-0.16 (-0.27, -0.05)	0.06	.004	.005
	Study PVT	0.07 (-0.04, 0.18)	0.06	.226	.181
	Test PVT	0.17 (0.01, 0.32)	0.08	.041	.041
**Difference in arousal**	General distress	-0.06 (-0.21, 0.10)	0.08	.450	.327
	Anhedonia	-0.29 (-0.43, -0.15)	0.07	<.001	<.001
	Anxious arousal	-0.32 (-0.54, -0.10)	0.11	.004	.005
	Age	0.02 (-0.08, 0.12)	0.05	.672	.448
	Sex	0.16 (0.06, 0.27)	0.05	.003	.005
	Study PVT	-0.32 (-0.51, -0.14)	0.09	<.001	<.001
	Test PVT	-0.01 (-0.15, 0.12)	0.07	.864	.494

Note. Adjusted q, two-stage controlling procedure for the false discovery rate.

Table S37

Standardized SEM Results of Internalizing Symptoms Measured by Bifactor S-1 Models Predicting Valence and Arousal Ratings Controlling for Age, Sex, and Psychomotor Vigilance Alertness

Outcome	Predictor	*ß with 95% CI*	*S.E.*	*p*	Adjusted q
**Valence for negative scenes**	General distress	0.05 (-0.08, 0.19)	0.07	.430	.399
	Anhedonia	0.14 (0.00, 0.27)	0.07	.047	.115
	Anxious arousal	0.16 (-0.06, 0.38)	0.11	.151	.185
	Age	-0.10 (-0.21, 0.01)	0.06	.086	.172
	Sex	-0.09 (-0.22, 0.03)	0.06	.148	.185
	Study PVT	0.23 (0.07, 0.38)	0.08	.005	.022
	Test PVT	0.07 (-0.07, 0.20)	0.07	.347	.347
**Valence for neutral scenes**	General distress	0.02 (-0.13, 0.17)	0.07	.773	.607
	Anhedonia	-0.34 (-0.47, -0.21)	0.07	<.001	<.001
	Anxious arousal	-0.09 (-0.25, 0.07)	0.08	.281	.300
	Age	0.04 (-0.07, 0.16)	0.06	.435	.399
	Sex	0.11 (-0.01, 0.22)	0.06	.065	.143
	Study PVT	0.14 (0.01, 0.26)	0.06	.036	.113
	Test PVT	-0.16 (-0.31, 0.00)	0.08	.044	.115
**Arousal for negative scenes**	General distress	-0.03 (-0.19, 0.13)	0.08	.706	.575
	Anhedonia	-0.12 (-0.26, 0.02)	0.07	.104	.185
	Anxious arousal	-0.14 (-0.39, 0.11)	0.13	.279	.300
	Age	0.09 (-0.02, 0.20)	0.06	.120	.185
	Sex	0.16 (0.04, 0.28)	0.06	.008	.029
	Study PVT	0.04 (-0.11, 0.20)	0.08	.593	.502
	Test PVT	-0.12 (-0.27, 0.04)	0.08	.134	.185
**Arousal for neutral scenes**	General distress	-0.11 (-0.24, 0.03)	0.07	.138	.185
	Anhedonia	0.27 (0.15, 0.38)	0.06	<.001	<.001
	Anxious arousal	0.27 (0.12, 0.42)	0.08	<.001	<.001
	Age	0.06 (-0.05, 0.17)	0.06	.286	.300
	Sex	-0.04 (-0.14, 0.06)	0.05	.454	.400
	Study PVT	0.49 (0.29, 0.69)	0.10	<.001	<.001
	Test PVT	-0.10 (-0.23, 0.03)	0.07	.129	.185

Note. Adjusted q, two-stage controlling procedure for the false discovery rate. Sex, coded as -1 for males and 1 for females. PVT, psychomotor vigilance test during encoding and memory sessions.

Table S38

Standardized SEM Results of Internalizing Symptoms Measured by Bifactor S-1 Models Predicting Specific Recognition Memory Controlling for Age, Sex, and Psychomotor Vigilance Alertness

Outcome for memory	Predictor	*ß with 95% CI*	*S.E.*	*p*	Adjusted q
**Negative objects**	General distress	0.01 (-0.12, 0.13)	0.06	.915	.824
	Anhedonia	0.07 (-0.07, 0.21)	0.07	.312	.541
	Anxious arousal	0.24 (0.07, 0.41)	0.09	.005	.036
	Age	0.01 (-0.11, 0.12)	0.06	.927	.824
	Sex	0.06 (-0.05, 0.17)	0.06	.302	.541
	Study PVT	-0.20 (-0.38, -0.02)	0.09	.028	.134
	Test PVT	-0.06 (-0.23, 0.10)	0.09	.453	.640
**Negative backgrounds**	General distress	0.07 (-0.07, 0.20)	0.07	.324	.541
	Anhedonia	0.01 (-0.13, 0.15)	0.07	.890	.824
	Anxious arousal	0.23 (0.08, 0.38)	0.07	.002	.036
	Age	0.02 (-0.10, 0.14)	0.06	.725	.824
	Sex	-0.01 (-0.13, 0.11)	0.06	.895	.824
	Study PVT	-0.11 (-0.23, 0.02)	0.06	.089	.267
	Test PVT	-0.04 (-0.16, 0.09)	0.07	.573	.724
**Neutral objects**	General distress	0.02 (-0.10, 0.14)	0.06	.739	.824
	Anhedonia	0.07 (-0.07, 0.21)	0.07	.338	.541
	Anxious arousal	0.17 (0.01, 0.34)	0.09	.040	.160
	Age	0.16 (0.05, 0.28)	0.06	.006	.036
	Sex	0.00 (-0.12, 0.12)	0.06	.993	.851
	Study PVT	-0.11 (-0.29, 0.06)	0.09	.202	.539
	Test PVT	-0.10 (-0.26, 0.06)	0.08	.229	.541
**Neutral backgrounds**	General distress	0.05 (-0.08, 0.18)	0.06	.440	.640
	Anhedonia	0.07 (-0.07, 0.21)	0.07	.307	.541
	Anxious arousal	0.21 (0.06, 0.36)	0.07	.005	.036
	Age	0.02 (-0.10, 0.14)	0.06	.797	.824
	Sex	0.02 (-0.10, 0.13)	0.06	.757	.824
	Study PVT	-0.17 (-0.34, 0.00)	0.09	.047	.161
	Test PVT	-0.04 (-0.18, 0.10)	0.07	.547	.724

Note. Adjusted q, two-stage controlling procedure for the false discovery rate. Sex, coded as -1 for males and 1 for females. PVT, psychomotor vigilance test during encoding and memory sessions.

Table S39

Standardized SEM Results of Internalizing Symptoms Measured by Bifactor S-1 Models Predicting Difference in Specific Recognition Memory Between Negative and Neutral Scene Components Controlling for Age, Sex, and Psychomotor Vigilance Alertness

Outcome	Predictor	*β with 95% CI*	*S.E.*	*p*	Adjusted q
**Difference in object memory**	General distress	-0.02 (-0.13, 0.10)	0.06	.787	.873
	Anhedonia	-0.01 (-0.15, 0.12)	0.07	.855	.873
	Anxious arousal	-0.11 (-0.25, 0.03)	0.07	.118	.433
	Age	-0.20 (-0.32, -0.08)	0.06	.001	.013
	Sex	0.08 (-0.04, 0.20)	0.06	.200	.433
	Study PVT	-0.13 (-0.26, 0.00)	0.06	.046	.299
	Test PVT	0.04 (-0.06, 0.14)	0.05	.409	.760
**Difference in background memory**	General distress	0.01 (-0.12, 0.14)	0.07	.873	.873
	Anhedonia	0.09 (-0.04, 0.22)	0.07	.187	.433
	Anxious arousal	0.02 (-0.10, 0.14)	0.06	.736	.873
	Age	0.00 (-0.13, 0.14)	0.07	.960	.891
	Sex	-0.03 (-0.15, 0.08)	0.06	.573	.873
	Study PVT	0.11 (-0.04, 0.26)	0.07	.149	.433
	Test PVT	0.01 (-0.08, 0.11)	0.05	.767	.873

Note. Adjusted q, two-stage controlling procedure for the false discovery rate.

Table S40

Standardized SEM Results of Internalizing Symptoms Measured by Bifactor S-1 Models Predicting Specific Negative and Neutral Memory Trade-Off Effects Controlling for Age, Sex, and Psychomotor Vigilance Alertness

Outcome	Predictor	*β with 95% CI*	*S.E.*	*p*	Adjusted q
**Negative memory trade-off**	General distress	0.05 (-0.08, 0.18)	0.07	.441	.719
	Anhedonia	-0.08 (-0.22, 0.06)	0.07	.269	.628
	Anxious arousal	-0.08 (-0.23, 0.07)	0.08	.319	.638
	Age	-0.01 (-0.13, 0.11)	0.06	.838	.902
	Sex	0.08 (-0.04, 0.20)	0.06	.206	.628
	Study PVT	-0.14 (-0.29, 0.01)	0.08	.060	.420
	Test PVT	-0.04 (-0.16, 0.07)	0.06	.462	.719
**Neutral memory trade-off**	General distress	0.03 (-0.10, 0.16)	0.07	.685	.872
	Anhedonia	-0.02 (-0.18, 0.14)	0.08	.787	.902
	Anxious arousal	0.03 (-0.10, 0.17)	0.07	.636	.872
	Age	0.18 (0.05, 0.31)	0.06	.006	.084
	Sex	-0.01 (-0.13, 0.11)	0.06	.902	.902
	Study PVT	0.06 (-0.04, 0.17)	0.05	.250	.628
	Test PVT	-0.07 (-0.17, 0.03)	0.05	.152	.628

Note. Adjusted q, two-stage controlling procedure for the false discovery rate.

Table S41

Standardized SEM Results of Internalizing Symptoms Measured by Bifactor S-1 Models Predicting Specific Full Memory Trade-Off Effects Controlling for Age, Sex, and Psychomotor Vigilance Alertness

Outcome	Predictor	*β with 95% CI*	*S.E.*	*p*	Adjusted q
**Full memory trade-off**	General distress	-0.02 (-0.15, 0.10)	0.06	.707	.715
	Anhedonia	-0.07 (-0.20, 0.07)	0.07	.337	.472
	Anxious arousal	-0.09 (-0.24, 0.06)	0.08	.244	.427
	Age	-0.15 (-0.28, -0.02)	0.06	.022	.077
	Sex	0.07 (-0.04, 0.20)	0.06	.220	.427
	Study PVT	-0.16 (-0.30, -0.03)	0.07	.017	.077
	Test PVT	0.02 (-0.08, 0.12)	0.05	.715	.715

Note. Adjusted q, two-stage controlling procedure for the false discovery rate.

view Only

Table S42

Standardized SEM Results of Internalizing Symptoms Measured by Bifactor S-1 Models Predicting Gist Recognition Memory Controlling for Age, Sex, and Psychomotor Vigilance Alertness

Outcome for memory	Predictor	*ß with 95% CI*	*S.E.*	*p*	Adjusted q
**Negative objects**	General distress	0.03 (-0.10, 0.17)	0.07	.614	.698
	Anhedonia	0.00 (-0.13, 0.14)	0.07	.943	.842
	Anxious arousal	-0.31 (-0.44, -0.17)	0.07	<.001	<.001
	Age	0.03 (-0.09, 0.14)	0.06	.672	.725
	Sex	0.09 (-0.02, 0.20)	0.06	.121	.336
	Study PVT	-0.14 (-0.27, -0.01)	0.07	.030	.150
	Test PVT	-0.07 (-0.21, 0.07)	0.07	.316	.564
**Negative backgrounds**	General distress	0.11 (-0.02, 0.24)	0.07	.099	.309
	Anhedonia	-0.02 (-0.15, 0.12)	0.07	.802	.781
	Anxious arousal	-0.31 (-0.47, -0.16)	0.08	<.001	<.001
	Age	0.05 (-0.07, 0.17)	0.06	.396	.582
	Sex	-0.01 (-0.13, 0.10)	0.06	.823	.781
	Study PVT	-0.09 (-0.20, 0.03)	0.06	.143	.357
	Test PVT	-0.04 (-0.15, 0.08)	0.06	.549	.668
**Neutral objects**	General distress	0.04 (-0.09, 0.17)	0.07	.546	.668
	Anhedonia	-0.07 (-0.20, 0.07)	0.07	.314	.564
	Anxious arousal	-0.18 (-0.36, 0.00)	0.09	.048	.200
	Age	0.13 (0.01, 0.25)	0.06	.027	.150
	Sex	0.02 (-0.09, 0.14)	0.06	.696	.725
	Study PVT	-0.13 (-0.31, 0.06)	0.10	.183	.416
	Test PVT	-0.02 (-0.20, 0.17)	0.10	.843	.781
**Neutral backgrounds**	General distress	0.05 (-0.06, 0.17)	0.06	.376	.582
	Anhedonia	-0.06 (-0.20, 0.08)	0.07	.378	.582
	Anxious arousal	-0.24 (-0.38, -0.09)	0.08	.002	.017
	Age	0.07 (-0.04, 0.19)	0.06	.219	.456
	Sex	0.05 (-0.07, 0.16)	0.06	.441	.613
	Study PVT	-0.13 (-0.28, 0.02)	0.08	.083	.296
	Test PVT	-0.03 (-0.15, 0.08)	0.06	.561	.668

Note. Adjusted q, two-stage controlling procedure for the false discovery rate. Sex, coded as -1 for males and 1 for females. PVT, psychomotor vigilance test during encoding and memory sessions.

Table S43

Standardized SEM Results of Internalizing Symptoms Measured by Bifactor S-1 Models Predicting Difference in Gist Recognition Memory Between Negative and Neutral Scene Components Controlling for Age, Sex, and Psychomotor Vigilance Alertness

Outcome	Predictor	*β with 95% CI*	*S.E.*	*p*	Adjusted q
**Difference in object memory**	General distress	-0.01 (-0.15, 0.14)	0.08	.937	.974
	Anhedonia	0.09 (-0.06, 0.23)	0.07	.244	.498
	Anxious arousal	-0.16 (-0.32, 0.00)	0.08	.043	.350
	Age	-0.12 (-0.23, 0.00)	0.06	.050	.350
	Sex	0.08 (-0.04, 0.20)	0.06	.179	.498
	Study PVT	-0.03 (-0.20, 0.15)	0.09	.744	.868
	Test PVT	-0.06 (-0.20, 0.07)	0.07	.363	.508
**Difference in background memory**	General distress	0.07 (-0.04, 0.19)	0.06	.210	.498
	Anhedonia	0.07 (-0.07, 0.22)	0.07	.304	.498
	Anxious arousal	-0.07 (-0.21, 0.07)	0.07	.320	.498
	Age	-0.04 (-0.15, 0.08)	0.06	.544	.692
	Sex	-0.09 (-0.21, 0.03)	0.06	.136	.498
	Study PVT	0.08 (-0.07, 0.24)	0.08	.290	.498
	Test PVT	0.00 (-0.14, 0.13)	0.07	.974	.974

Note. Adjusted q, two-stage controlling procedure for the false discovery rate.

Table S44

Standardized SEM Results of Internalizing Symptoms Measured by Bifactor S-1 Models Predicting Gist Negative and Neutral Memory Trade-Off Effects Controlling for Age, Sex, and Psychomotor Vigilance Alertness

Outcome	Predictor	*β with 95% CI*	*S.E.*	*p*	Adjusted q
**Negative memory trade-off**	General distress	0.06 (-0.08, 0.20)	0.07	.401	.936
	Anhedonia	0.02 (-0.12, 0.15)	0.07	.780	.967
	Anxious arousal	-0.08 (-0.23, 0.07)	0.07	.300	.936
	Age	-0.02 (-0.13, 0.10)	0.06	.757	.967
	Sex	0.12 (0.00, 0.24)	0.06	.050	.700
	Study PVT	-0.09 (-0.22, 0.04)	0.07	.172	.803
	Test PVT	-0.05 (-0.16, 0.06)	0.06	.336	.936
**Neutral memory trade-off**	General distress	-0.01 (-0.14, 0.13)	0.07	.938	.967
	Anhedonia	-0.03 (-0.17, 0.12)	0.07	.711	.967
	Anxious arousal	0.04 (-0.12, 0.20)	0.08	.619	.967
	Age	0.09 (-0.03, 0.21)	0.06	.146	.803
	Sex	-0.01 (-0.13, 0.11)	0.06	.856	.967
	Study PVT	0.00 (-0.14, 0.15)	0.07	.967	.967
	Test PVT	-0.01 (-0.19, 0.17)	0.09	.942	.967

Note. Adjusted q, two-stage controlling procedure for the false discovery rate.

Table S45

Standardized SEM Results of Internalizing Symptoms Measured by Bifactor S-1 Models Predicting Gist Full Memory Trade-Off Effects Controlling for Age, Sex, and Psychomotor Vigilance Alertness

Outcome	Predictor	*β with 95% CI*	*S.E.*	*p*	Adjusted q
**Full memory trade-off**	General distress	0.05 (-0.09, 0.18)	0.07	.515	.540
	Anhedonia	0.05 (-0.09, 0.18)	0.07	.484	.540
	Anxious arousal	-0.10 (-0.25, 0.06)	0.08	.215	.502
	Age	-0.08 (-0.20, 0.04)	0.06	.175	.502
	Sex	0.11 (-0.01, 0.23)	0.06	.067	.469
	Study PVT	-0.07 (-0.22, 0.08)	0.08	.363	.540
	Test PVT	-0.04 (-0.18, 0.09)	0.07	.540	.540

Note. Adjusted q, two-stage controlling procedure for the false discovery rate.

view Only

Structure Equation Modeling Controlling for Encoding Ratings

Table S46

Standardized SEM Results of Internalizing Symptoms Measured by Bifactor S-1 Models Predicting Specific Recognition Memory Controlling for Differences in Valence and Arousal Ratings

Outcome for memory	Predictor	*ß with 95% CI*	*S.E.*	*p*	Adjusted q
**Negative objects**	General distress	0.04 (-0.07, 0.16)	0.06	.485	.601
	Anhedonia	0.01 (-0.14, 0.16)	0.08	.894	.753
	Anxious arousal	-0.19 (-0.33, -0.05)	0.07	.009	.036
	Valence difference	-0.08 (-0.31, 0.15)	0.12	.492	.601
	Arousal difference	0.36 (0.13, 0.60)	0.12	.002	.016
**Negative backgrounds**	General distress	0.09 (-0.04, 0.22)	0.07	.197	.394
	Anhedonia	0.07 (-0.07, 0.22)	0.07	.316	.460
	Anxious arousal	-0.19 (-0.32, -0.06)	0.07	.005	.027
	Valence difference	0.07 (-0.17, 0.30)	0.12	.565	.601
	Arousal difference	0.13 (-0.12, 0.38)	0.13	.314	.460
**Neutral objects**	General distress	0.02 (-0.09, 0.13)	0.06	.744	.661
	Anhedonia	0.04 (-0.11, 0.18)	0.07	.601	.601
	Anxious arousal	-0.11 (-0.24, 0.02)	0.07	.092	.210
	Valence difference	0.00 (-0.17, 0.18)	0.09	.964	.771
	Arousal difference	0.34 (0.18, 0.51)	0.09	<.001	<.001
**Neutral backgrounds**	General distress	0.07 (-0.05, 0.20)	0.06	.237	.421
	Anhedonia	0.03 (-0.12, 0.17)	0.07	.697	.656
	Anxious arousal	-0.15 (-0.28, -0.02)	0.07	.027	.086
	Valence difference	0.06 (-0.14, 0.27)	0.10	.551	.601
	Arousal difference	0.20 (0.00, 0.41)	0.10	.048	.128

Note. Adjusted q, two-stage controlling procedure for the false discovery rate.

Table S47

Standardized SEM Results of Internalizing Symptoms Measured by Bifactor S-1 Models Predicting Difference in Specific Recognition Memory Between Negative and Neutral Scenes Controlling for Differences in Valence and Arousal Ratings

Outcome for memory	Predictor	*ß with 95% CI*	*S.E.*	*p*	Adjusted q
**Difference in object memory**	General distress	0.04 (-0.08, 0.15)	0.06	.548	.990
	Anhedonia	-0.03 (-0.18, 0.11)	0.07	.651	.990
	Anxious arousal	-0.12 (-0.27, 0.02)	0.08	.101	.505
	Valence difference	-0.12 (-0.35, 0.12)	0.12	.334	.990
	Arousal difference	0.05 (-0.22, 0.33)	0.14	.709	.990
**Difference in background memory**	General distress	0.00 (-0.13, 0.13)	0.07	.990	.990
	Anhedonia	0.05 (-0.09, 0.19)	0.07	.512	.990
	Anxious arousal	-0.01 (-0.14, 0.13)	0.07	.896	.990
	Valence difference	-0.01 (-0.17, 0.16)	0.08	.939	.990
	Arousal difference	-0.14 (-0.29, 0.03)	0.08	.098	.505

Note. Adjusted q, two-stage controlling procedure for the false discovery rate.

Only

Table S48

Standardized SEM Results of Internalizing Symptoms Measured by Bifactor S-1 Models Predicting Specific Negative and Neutral Memory Trade-Off Effects Controlling for Differences in Valence and Arousal

Outcome for memory	Predictor	*ß with 95% CI*	*S.E.*	*p*	Adjusted q
**Negative memory trade-off**	General distress	0.03 (-0.10, 0.16)	0.06	.648	.648
	Anhedonia	-0.05 (-0.20, 0.10)	0.08	.484	.588
	Anxious arousal	-0.06 (-0.21, 0.09)	0.07	.431	.588
	Valence difference	-0.16 (-0.32, 0.00)	0.08	.045	.202
	Arousal difference	0.32 (0.18, 0.46)	0.07	<.001	<.001
**Neutral memory trade-off**	General distress	0.07 (-0.05, 0.18)	0.06	.279	.588
	Anhedonia	0.01 (-0.15, 0.17)	0.08	.899	.809
	Anxious arousal	0.05 (-0.10, 0.19)	0.07	.523	.588
	Valence difference	-0.07 (-0.24, 0.11)	0.09	.439	.588
	Arousal difference	0.18 (-0.01, 0.38)	0.10	.069	.207

Note. Adjusted q, two-stage controlling procedure for the false discovery rate.

Only

Table S49

Standardized SEM Results of Internalizing Symptoms Measured by Bifactor S-1 Models Predicting Specific Full Memory Trade-Off Effects Controlling for Differences in Valence and Arousal Ratings

Outcome for memory	Predictor	*ß with 95% CI*	*S.E.*	*p*	Adjusted q
**Full memory trade-off**	General distress	0.03 (-0.09, 0.14)	0.06	.671	.671
	Anhedonia	-0.06 (-0.21, 0.09)	0.08	.468	.585
	Anxious arousal	-0.09 (-0.25, 0.07)	0.08	.278	.585
	Valence difference	-0.08 (-0.27, 0.10)	0.10	.384	.585
	Arousal difference	0.13 (-0.07, 0.33)	0.10	.206	.585

Note. Adjusted q, two-stage controlling procedure for the false discovery rate.

Review Only

Table S50

Standardized SEM Results of Internalizing Symptoms Measured by Bifactor S-1 Models Predicting Gist Recognition Memory Controlling for Differences in Valence and Arousal Ratings

Outcome for memory	Predictor	*ß with 95% CI*	*S.E.*	*p*	Adjusted q
**Negative objects**	General distress	0.06 (-0.07, 0.18)	0.06	.390	.451
	Anhedonia	0.04 (-0.11, 0.18)	0.07	.605	.547
	Anxious arousal	-0.27 (-0.40, -0.14)	0.07	<.001	<.001
	Valence difference	-0.17 (-0.37, 0.03)	0.10	.093	.140
	Arousal difference	0.34 (0.14, 0.54)	0.10	.001	.004
**Negative backgrounds**	General distress	0.12 (0.00, 0.25)	0.06	.049	.092
	Anhedonia	0.03 (-0.11, 0.17)	0.07	.681	.547
	Anxious arousal	-0.25 (-0.38, -0.12)	0.07	<.001	<.001
	Valence difference	-0.06 (-0.26, 0.14)	0.10	.531	.531
	Arousal difference	0.25 (0.05, 0.46)	0.11	.017	.043
**Neutral objects**	General distress	0.05 (-0.07, 0.17)	0.06	.391	.451
	Anhedonia	-0.03 (-0.18, 0.13)	0.08	.729	.547
	Anxious arousal	-0.14 (-0.28, 0.01)	0.07	.068	.113
	Valence difference	-0.07 (-0.28, 0.14)	0.11	.525	.531
	Arousal difference	0.31 (0.12, 0.50)	0.10	.001	.004
**Neutral backgrounds**	General distress	0.06 (-0.05, 0.18)	0.06	.266	.363
	Anhedonia	0.03 (-0.12, 0.17)	0.07	.724	.547
	Anxious arousal	-0.17 (-0.29, -0.04)	0.06	.008	.024
	Valence difference	0.04 (-0.14, 0.22)	0.09	.622	.547
	Arousal difference	0.21 (0.02, 0.41)	0.10	.028	.060

Note. Adjusted q, two-stage controlling procedure for the false discovery rate.

Table S51

Standardized SEM Results of Internalizing Symptoms Measured by Bifactor S-1 Models Predicting Difference in Gist Recognition Memory Between Negative and Neutral Scenes Controlling for Differences in Valence and Arousal

Outcome for memory	Predictor	*ß with 95% CI*	*S.E.*	*p*	Adjusted q
**Difference in object memory**	General distress	0.01 (-0.15, 0.16)	0.08	.922	.995
	Anhedonia	0.08 (-0.08, 0.24)	0.08	.324	.540
	Anxious arousal	-0.18 (-0.37, 0.01)	0.10	.063	.350
	Valence difference	-0.13 (-0.31, 0.05)	0.09	.153	.382
	Arousal difference	0.06 (-0.16, 0.28)	0.11	.593	.847
**Difference in background memory**	General distress	0.08 (-0.04, 0.19)	0.06	.182	.382
	Anhedonia	0.00 (-0.15, 0.15)	0.08	.995	.995
	Anxious arousal	-0.09 (-0.22, 0.04)	0.07	.191	.382
	Valence difference	-0.17 (-0.35, 0.01)	0.09	.070	.350
	Arousal difference	0.03 (-0.14, 0.20)	0.09	.739	.924

Note. Adjusted q, two-stage controlling procedure for the false discovery rate.

For Review Only

Table S52

Standardized SEM Results of Internalizing Symptoms Measured by Bifactor S-1 Models Predicting Gist Negative and Neutral Memory Trade-Off Effects Controlling for Differences in Valence and Arousal Ratings

Outcome for memory	Predictor	*ß with 95% CI*	*S.E.*	*p*	Adjusted q
**Negative memory trade-off**	General distress	0.04 (-0.09, 0.18)	0.07	.530	.757
	Anhedonia	0.02 (-0.12, 0.16)	0.07	.791	.934
	Anxious arousal	-0.10 (-0.25, 0.04)	0.07	.173	.432
	Valence difference	-0.15 (-0.30, 0.00)	0.08	.058	.290
	Arousal difference	0.19 (0.03, 0.34)	0.08	.018	.180
**Neutral memory trade-off**	General distress	0.01 (-0.13, 0.14)	0.07	.934	.934
	Anhedonia	-0.06 (-0.21, 0.09)	0.07	.431	.718
	Anxious arousal	0.02 (-0.15, 0.18)	0.09	.850	.934
	Valence difference	-0.13 (-0.36, 0.10)	0.12	.271	.542
	Arousal difference	0.15 (-0.05, 0.36)	0.10	.137	.432

Note. Adjusted q, two-stage controlling procedure for the false discovery rate.

Only

Table S53

Standardized SEM Results of Internalizing Symptoms Measured by Bifactor S-1 Models Predicting Gist Full Memory Trade-Off Effects Controlling for Differences in Valence and Arousal Ratings

Outcome for memory	Predictor	*ß with 95% CI*	*S.E.*	*p*	Adjusted q
**Full memory trade-off**	General distress	0.03 (-0.11, 0.17)	0.07	.639	.803
	Anhedonia	0.06 (-0.08, 0.21)	0.07	.382	.803
	Anxious arousal	-0.10 (-0.28, 0.08)	0.09	.272	.803
	Valence difference	-0.03 (-0.22, 0.17)	0.10	.803	.803
	Arousal difference	0.04 (-0.16, 0.24)	0.10	.721	.803

Note. Adjusted q, two-stage controlling procedure for the false discovery rate.

Review Only

Structure Equation Modeling Excluding Nappers

Table S54

Standardized Multigroup SEM Results of Internalizing Symptoms Measured by Bifactor S-1 Models Predicting Difference in Valence and Arousal Ratings in the Wake and Sleep Conditions Excluding 30 Participants Who Napped During Retention Intervals

Outcome	Predictor	*β with 95% CI*	*S.E.*	*p*	Adjusted q
		**Daytime Wake**			
**Difference in valence**	General distress	-0.09 (-0.29, 0.11)	0.10	.363	.262
	Anhedonia	-0.39 (-0.58, -0.21)	0.10	<.001	<.001
	Anxious arousal	-0.33 (-0.52, -0.14)	0.10	.001	.001
**Difference in arousal**	General distress	-0.10 (-0.33, 0.12)	0.12	.374	.262
	Anhedonia	-0.19 (-0.39, 0.02)	0.10	.075	.075
	Anxious arousal	-0.42 (-0.63, -0.21)	0.10	<.001	<.001
		**Nighttime Sleep**			
**Difference in valence**	General distress	0.16 (-0.01, 0.34)	0.09	.060	.070
	Anhedonia	-0.33 (-0.52, -0.13)	0.10	.001	.001
	Anxious arousal	0.12 (-0.08, 0.31)	0.10	.242	.212
**Difference in arousal**	General distress	0.04 (-0.13, 0.21)	0.09	.649	.413
	Anhedonia	-0.45 (-0.64, -0.26)	0.10	<.001	<.001
	Anxious arousal	-0.02 (-0.21, 0.16)	0.10	.815	.475

Note. Adjusted q, two-stage controlling procedure for the false discovery rate.

Table S55

Standardized Multigroup SEM Results of Internalizing Symptoms Measured by Bifactor S-1 Models Predicting Valence and Arousal Ratings in the Wake and Sleep Conditions Excluding 30 Participants Who Napped During Retention Intervals

Outcome	Predictor	*β with 95% CI*	*S.E.*	*p*	Adjusted q
		**Daytime Wake**			
**Valence for negative scenes**	General distress	-0.05 (-0.25, 0.15)	0.10	.632	.600
	Anhedonia	0.14 (-0.11, 0.38)	0.12	.274	.325
	Anxious arousal	0.24 (-0.01, 0.48)	0.12	.057	.135
**Valence for neutral scenes**	General distress	-0.20 (-0.38, -0.02)	0.09	.029	.092
	Anhedonia	-0.46 (-0.61, -0.31)	0.07	<.001	<.001
	Anxious arousal	-0.25 (-0.37, -0.13)	0.06	<.001	<.001
**Arousal for negative scenes**	General distress	-0.04 (-0.27, 0.19)	0.12	.737	.602
	Anhedonia	-0.13 (-0.34, 0.09)	0.11	.243	.325
	Anxious arousal	-0.29 (-0.61, 0.04)	0.16	.081	.171
**Arousal for neutral scenes**	General distress	0.13 (-0.10, 0.36)	0.12	.261	.325
	Anhedonia	0.16 (-0.04, 0.36)	0.10	.113	.195
	Anxious arousal	0.31 (0.09, 0.53)	0.11	.005	.024
		**Nighttime Sleep**			
**Valence for negative scenes**	General distress	-0.02 (-0.18, 0.13)	0.08	.761	.602
	Anhedonia	0.14 (-0.07, 0.35)	0.11	.204	.298
	Anxious arousal	-0.10 (-0.24, 0.05)	0.07	.193	.298
**Valence for neutral scenes**	General distress	0.19 (0.01, 0.38)	0.09	.040	.109
	Anhedonia	-0.28 (-0.49, -0.07)	0.10	.008	.030
	Anxious arousal	0.04 (-0.16, 0.25)	0.10	.686	.602
**Arousal for negative scenes**	General distress	0.09 (-0.10, 0.28)	0.10	.382	.403
	Anhedonia	-0.17 (-0.38, 0.03)	0.11	.100	.190
	Anxious arousal	0.08 (-0.12, 0.28)	0.10	.446	.446
**Arousal for neutral scenes**	General distress	0.03 (-0.13, 0.19)	0.08	.705	.602
	Anhedonia	0.42 (0.25, 0.60)	0.09	<.001	<.001
	Anxious arousal	0.11 (-0.11, 0.32)	0.11	.325	.363

Note. Adjusted q, two-stage controlling procedure for the false discovery rate.

Table S56

Standardized Multigroup SEM Results of Internalizing Symptoms Measured by Bifactor S-1 Models Predicting Specific Recognition memory in the Wake and Sleep Conditions Excluding 30 Participants Who Napped During Retention Intervals

Outcome	Predictor	*β with 95% CI*	*S.E.*	*p*	Adjusted q
		**Daytime Wake**			
**Negative objects**	General distress	0.05 (-0.13, 0.24)	0.09	.559	.696
	Anhedonia	0.00 (-0.22, 0.23)	0.12	.981	.820
	Anxious arousal	-0.28 (-0.46, -0.11)	0.09	.002	.010
**Negative backgrounds**	General distress	0.09 (-0.10, 0.27)	0.09	.357	.537
	Anhedonia	-0.07 (-0.25, 0.12)	0.09	.476	.635
	Anxious arousal	-0.29 (-0.47, -0.12)	0.09	.001	.007
**Neutral objects**	General distress	-0.03 (-0.20, 0.14)	0.09	.717	.787
	Anhedonia	-0.01 (-0.23, 0.21)	0.11	.910	.820
	Anxious arousal	-0.28 (-0.42, -0.13)	0.07	<.001	<.001
**Neutral backgrounds**	General distress	0.10 (-0.09, 0.29)	0.10	.314	.537
	Anhedonia	-0.16 (-0.35, 0.03)	0.10	.106	.334
	Anxious arousal	-0.27 (-0.42, -0.12)	0.08	<.001	<.001
		**Nighttime Sleep**			
**Negative objects**	General distress	0.08 (-0.07, 0.24)	0.08	.292	.537
	Anhedonia	-0.10 (-0.31, 0.12)	0.11	.376	.537
	Anxious arousal	-0.03 (-0.24, 0.19)	0.11	.825	.787
**Negative backgrounds**	General distress	0.15 (-0.04, 0.34)	0.10	.117	.334
	Anhedonia	0.06 (-0.15, 0.26)	0.11	.592	.696
	Anxious arousal	-0.10 (-0.32, 0.11)	0.11	.354	.537
**Neutral objects**	General distress	0.11 (-0.05, 0.26)	0.08	.168	.420
	Anhedonia	-0.12 (-0.31, 0.07)	0.10	.215	.478
	Anxious arousal	-0.04 (-0.28, 0.20)	0.12	.749	.787
**Neutral backgrounds**	General distress	0.13 (-0.03, 0.28)	0.08	.112	.334
	Anhedonia	0.02 (-0.19, 0.23)	0.11	.826	.787
	Anxious arousal	0.00 (-0.25, 0.25)	0.13	.984	.820

Note. Adjusted q, two-stage controlling procedure for the false discovery rate.

Table S57

Standardized Multigroup SEM Results of Internalizing Symptoms Measured by Bifactor S-1 Models Predicting Difference in Specific Recognition Memory Between Negative and Neutral Scene Components in the Wake and Sleep Conditions Excluding 30 Participants Who Napped During Retention Intervals

Outcome	Predictor	*β with 95% CI*	*S.E.*	*p*	Adjusted q
		**Daytime Wake**			
**Difference in object memory**	General distress	0.12 (-0.06, 0.31)	0.10	.189	.910
	Anhedonia	0.02 (-0.18, 0.22)	0.10	.866	.910
	Anxious arousal	-0.05 (-0.22, 0.12)	0.09	.560	.910
**Difference in background memory**	General distress	-0.03 (-0.22, 0.16)	0.10	.727	.910
	Anhedonia	0.14 (-0.07, 0.34)	0.11	.201	.910
	Anxious arousal	0.05 (-0.10, 0.20)	0.08	.522	.910
		**Nighttime Sleep**			
**Difference in object memory**	General distress	-0.02 (-0.17, 0.13)	0.08	.769	.910
	Anhedonia	0.02 (-0.18, 0.22)	0.10	.844	.910
	Anxious arousal	0.01 (-0.19, 0.22)	0.10	.892	.910
**Difference in background memory**	General distress	0.01 (-0.18, 0.21)	0.10	.910	.910
	Anhedonia	0.04 (-0.14, 0.21)	0.09	.681	.910
	Anxious arousal	-0.12 (-0.36, 0.13)	0.12	.346	.910

Note. Adjusted q, two-stage controlling procedure for the false discovery rate.

Table S58

Standardized Multigroup SEM Results of Internalizing Symptoms Measured by Bifactor S-1 Models Predicting Specific Negative and Neutral Memory Trade-Off Effects in the Wake and Sleep Conditions Excluding 30 Participants Who Napped During Retention Intervals

Outcome	Predictor	*β with 95% CI*	*S.E.*	*p*	Adjusted q
		**Daytime Wake**			
**Negative memory trade-off**	General distress	-0.01 (-0.18, 0.15)	0.09	.890	.929
	Anhedonia	0.06 (-0.17, 0.29)	0.12	.624	.929
	Anxious arousal	-0.09 (-0.28, 0.11)	0.10	.386	.926
**Neutral memory trade-off**	General distress	-0.15 (-0.33, 0.02)	0.09	.079	.436
	Anhedonia	0.16 (-0.09, 0.42)	0.13	.212	.636
	Anxious arousal	-0.01 (-0.13, 0.12)	0.06	.929	.929
		**Nighttime Sleep**			
**Negative memory trade-off**	General distress	-0.04 (-0.24, 0.17)	0.10	.731	.929
	Anhedonia	-0.16 (-0.34, 0.03)	0.10	.105	.436
	Anxious arousal	0.06 (-0.15, 0.28)	0.11	.571	.929
**Neutral memory trade-off**	General distress	-0.01 (-0.18, 0.16)	0.09	.905	.929
	Anhedonia	-0.17 (-0.37, 0.04)	0.10	.109	.436
	Anxious arousal	-0.04 (-0.32, 0.23)	0.14	.754	.929

Note. Adjusted q, two-stage controlling procedure for the false discovery rate.

Table S59

Standardized Multigroup SEM Results of Internalizing Symptoms Measured by Bifactor S-1 Models Predicting Specific Full Trade-Off Effects in the Wake and Sleep Conditions Excluding 30 Participants Who Napped During Retention Intervals

Outcome	Predictor	*β with 95% CI*	*S.E.*	*p*	Adjusted q
		**Daytime Wake**			
**Full memory trade-off**	General distress	0.12 (-0.07, 0.30)	0.09	.223	.702
	Anhedonia	-0.08 (-0.29, 0.14)	0.11	.468	.702
	Anxious arousal	-0.07 (-0.26, 0.12)	0.10	.460	.702
		**Nighttime Sleep**			
**Full memory trade-off**	General distress	-0.02 (-0.20, 0.15)	0.09	.791	.919
	Anhedonia	-0.01 (-0.20, 0.18)	0.10	.919	.919
	Anxious arousal	0.09 (-0.15, 0.33)	0.12	.466	.702

Note. Adjusted q, two-stage controlling procedure for the false discovery rate.

Review Only
